# Biochemical and Structural Diversification of C_4_ Photosynthesis in Tribe Zoysieae (Poaceae)

**DOI:** 10.3390/plants12234049

**Published:** 2023-11-30

**Authors:** Nuria K. Koteyeva, Elena V. Voznesenskaya, Varsha S. Pathare, Tatyana A. Borisenko, Peter M. Zhurbenko, Grigory A. Morozov, Gerald E. Edwards

**Affiliations:** 1 Laboratory of Anatomy and Morphology, Komarov Botanical Institute of Russian Academy of Sciences, 197376 St. Petersburg, Russia; st075629@student.spbu.ru; 2 Institute for Genomic Biology, University of Illinois at Urbana-Champaign, Champaign, IL 61801, USA; varsha.pathare@wsu.edu; 3 Faculty of Biology, St. Petersburg State University, 199034 St. Petersburg, Russia; 4 Laboratory of Biosystematics and Cytology, Komarov Botanical Institute of Russian Academy of Sciences, 197376 St. Petersburg, Russia; pj_28@mail.ru; 5 Chair of Medical Biology, North-Western State Medical University named after I.I. Mechnikov, 191015 St. Petersburg, Russia; dartost1@rambler.ru; 6 School of Biological Sciences, Washington State University, Pullman, WA 99164-4236, USA; edwardsg@wsu.edu

**Keywords:** C_4_ photosynthesis, decarboxylases, evolution, Kranz anatomy, Poaceae

## Abstract

C_4_ photosynthesis has evolved independently multiple times in grass lineages with nine anatomical and three biochemical subtypes. Chloridoideae represents one of the separate events and contains species of two biochemical subtypes, NAD-ME and PEP-CK. Assessment of C_4_ photosynthesis diversification is limited by species sampling. In this study, the biochemical subtypes together with anatomical leaf traits were analyzed in 19 species to reveal the evolutionary scenario for diversification of C_4_ photosynthesis in tribe Zoysieae (Chloridoideae). The effect of habitat on anatomical and biochemical diversification was also evaluated. The results for the 19 species studied indicate that 11 species have only NAD-ME as a decarboxylating enzyme, while eight species belong to the PEP-CK subtype. Leaf anatomy corresponds to the biochemical subtype. Analysis of Zoysieae phylogeny indicates multiple switches between PEP-CK and NAD-ME photosynthetic subtypes, with PEP-CK most likely as the ancestral subtype, and with multiple independent PEP-CK decarboxylase losses and its secondary acquisition. A strong correlation was detected between C_4_ biochemical subtypes studied and habitat annual precipitation wherein NAD-ME species are confined to drier habitats, while PEP-CK species prefer humid areas. Structural adaptations to arid climate include increases in leaf thickness and interveinal distance. Our analysis suggests that multiple loss of PEP-CK decarboxylase could have been driven by climate aridization followed by continued adaptive changes in leaf anatomy.

## 1. Introduction

C_4_ photosynthesis requires spatial separation of two processes: carbon assimilation via PEP-carboxylase and carbon reduction by Calvin cycle after the decarboxylation of primary C_4_ products. This increases CO_2_ concentration around Rubisco, minimizing photorespiration. This is achieved via biochemical and anatomical specialization of the photosynthetic apparatus. The majority of C_4_ plants use the Kranz anatomy system, consisting of dual chlorenchyma layers, mesophyll (M), and bundle sheath (BS). The primary capturing of atmospheric CO_2_ occurs in M cells with formation of C_4_ acids, which are then decarboxylated and assimilated via the Calvin cycle in BS cells [1,2]. Multiple types of Kranz anatomy are recognized in different lineages based on the position of M and BS in relation to vascular bundles, position, and the number of organelles in BS; ultrastructural differentiation of chloroplasts and mitochondria between M and BS; the presence or absence of suberin deposition; and occurrence and position of specialized tissues (for example, mestome sheath in monocots or water storage in dicots) [3].

Three biochemical subtypes are recognized based on the predominant decarboxylases: NAD-ME, NADP-ME and PEP-CK [4,5]. All three occur only in grasses with the PEP-CK subtype found exceptionally in Panicoideae and Chloridoideae subfamilies [6]. In many C_4_ plants, different decarboxylases co-exist with varying levels of expression and activity. Usually NAD-ME complements the NADP-ME and PEP-CK pathways [7,8,9], and PEP-CK can contribute to the NADP-ME cycle [9,10,11]. According to transcript studies and Western blotting, some trace amounts of PEP-CK have been found in dicots and monocots with the NAD-ME biochemical subtype [12,13,14]; however, enzymatic assays suggest there is little capacity for PEP-CK to contribute to C_4_ photosynthesis in NAD-ME plants [12]. The flexibility in the combination of decarboxylases directly relates to energy requirements for plant metabolism, and finally to acclimation [15].

In general, biochemical subtypes are characterized by specific anatomical traits [3,16,17]. NAD-ME species usually have centripetal distribution of BS chloroplasts and numerous mitochondria, with deficiency in grana development in M chloroplasts and well-developed grana in BS chloroplasts. BS chloroplasts in NADP-ME species are distributed centrifugally and are nearly agranal, while M chloroplasts are granal. The PEP-CK subtype varies in BS chloroplast distribution with a tendency to be centrifugal, while M and BS chloroplasts have nearly equal grana development. However, such a classification is not strictly fixed, having numerous exceptions among dicots and grasses [18]. As a well-known example, *Eragrostis* species with centripetal BS organelle positioning (defined as “NAD-ME-like” C_4_ anatomy in [19]) and centrifugal BS organelle positioning (defined as “PEP-CK-like” anatomy in [19]) were biochemically recognized as the NAD-ME subtype [12]. Therefore, anatomical clues can be misleading in biochemical subtype identification, but they are important for the detailed description of C_4_ metabolism and ecological adaptations of species.

Despite C_4_ photosynthesis being a complex with multigene traits, it has evolved independently about 62 times in 19 plant lineages, including up to 24 origins in grasses [20,21]. Within most lines, further improvements, diversifications and reversions have occurred under selective pressure, leading to diversity in biochemical and anatomical C_4_ types and the ecological niches they occupy [22,23]. The main problem in the study of C_4_ diversifications is species sampling. Herbarium material is sufficient to recognize C_4_ plants via carbon isotope analyses. However, to determine the biochemical, physiological, and anatomical features of the C_4_ type, it is necessary to have living plants. Among 12074 known grass species [24], about 5044 species are recognized as having C_4_ metabolism [20]; however, only for a limited number of species is the biochemical subtype confirmed using appropriate methods. So, increasing sampling is crucial for revealing the evolutionary scenario of C_4_ diversification under the selective pressure responsible for the transitions and adaptive changes. 

Tribe Zoysieae is one of the grass lineages that contains both PEP-CK and NAD-ME subtypes and different anatomical types of C_4_ photosynthesis, which makes it a suitable model for the study of C_4_ evolutionary diversification. The tribe contains 244 species in two subtribes and 4 genera [24]. Some 14 species among them have identified biochemical subtype confirmed by analyses for three decarboxylases [4,5,9,12,16], and about 17 species have been classified based solely on analyses of leaf anatomy or on only one decarboxylase (PEP-CK) via Western blots, activity, or transcripts [25,26,27,28,29]. In total, among the studied species of Zoysieae, 24 PEP-CK versus 7 NAD-ME C_4_ subtypes have been recognized. Additionally several species have been used for study of the features of C_4_ metabolism and adaptive reactions [30,31,32], and some species are economically important as turfgrass [20]. Subtribe Zoysiinae contains two genera [33,34]: *Zoysia* classified as PEP-CK (*Z. japonica*, [4]), and *Urochondra,* which is NAD-ME [12]. Subtribe Sporobolinae contains two genera [33,34]: *Psilolemma,* which is C_4_ with an unknown biochemical subtype [35], and *Sporobolus,* which contains PEP-CK and NAD-ME species. 

The focus of the current study was to detect or confirm the already known C_4_ anatomical and biochemical subtypes in 19 species from the tribe Zoysieae (Chloridoideae, Poaceae). By using a phylogenetic analysis and mapping the studied traits, this study aimed to reveal the evolutionary scenario for the diversification of C_4_ subtypes with an increase in species sampling covering most sections of the tribe. The goal of this study is to explore the relationship of biochemical and anatomical traits with habitat features to infer the adaptive values of decarboxylating enzyme switches and subsequent anatomical modifications. 

## 2. Results

### 2.1. Western Blotting

**Figure 1:**
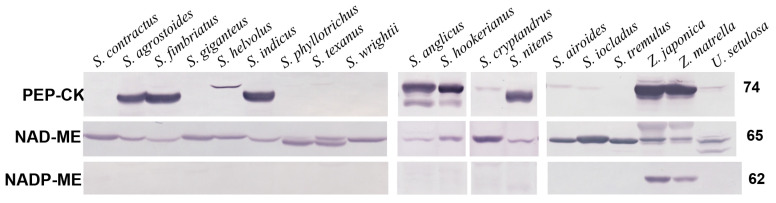
Western blots for three decarboxylases from total proteins extracted from leaves of 19 Zoysieae species. Blots were probed with antibodies raised against PEP-CK, NAD-ME, and NADP-ME: representative Western blots are presented, showing detection of each protein. The originals were modified for alignment according to species or to avoid replicate species (vertical lines); there were no selective changes in the mass or densities of bands on the membrane. The molecular mass is indicated to the right of the blots.

Immunoblots are shown for three decarboxylases, NAD-ME, PEP-CK, and NADP-ME, from soluble proteins extracted from leaves of 19 Zoysieae species; biochemical subtypes were determined (13 species) or confirmed (6 species, Supplementary Table S3) where known. All species studied have labelling for NAD-ME with different, but prominent intensities (Figure 1; Supplementary Figure S1). Eight species have high labelling for PEP-CK. *S. anglicus* and *S. hookerianus* show two bands with a major band at 74 kDa and a minor band at 65 kDa. Two *Zoysia* species have a single band at 74 kDa, and *S. agrostoides*, *S. fimbriatus*, *S. indicus*, and *S. nitens* have single bands at ~69 kDa. In *S. helvolus, S. cryptandrus, S. airoides*, *S. iocladus*, and *U. setulosa,* the labelling for PEP-CK is barely detectable at ~74 kDa. There is sufficient labelling with NADP-ME in only two *Zoysia* species (Figure 1; Supplementary Figure S1). 

Analyses of the relative band densities are presented in Table 1 as a percentage of band intensity level relative to the band intensity in reference species (in *S. anglicus* for PEP-CK, in *S. airoides* for NAD-ME, and in *Zea mays* for NADP-ME). Species that have NAD-ME as the only or predominant decarboxylase (NAD-ME species) have a 1.9 times difference in the density of NAD-ME labelling. The density was the lowest in *U. setulosa* (73% of *S. airoides*) and the highest in *S. ioclados* and *S. texanus* (about 140%). *Zoysia* species with PEP-CK as a predominant decarboxylase show high NAD-ME density with values comparable with NAD-ME species, while in the rest of *Sporobolus* species with high PEP-CK expression, NAD-ME was less prominent than in NAD-ME species, being the lowest in *S. nitens* (25%). On average, NAD-ME species have twice as much NAD-ME decarboxylase expression as PEP-CK species (Table 1). 

The amount of PEP-CK varies by about four times among the eight species that have this decarboxylase as a predominate form. Relative to *S. anglicus,* the highest intensity of labelling was found in *Zoysia* species (with intensity in *Z. japonica* being twice as high); a similar amount was shown in *S. hookerianus*, and the lowest labelling was in the rest of the *Sporobolus* species. Species with a very small amount of PEP-CK (*S. airoides*, *S. cryptandrus, S. helvolus*, *S. ioclados,* and *U. setulosa*) demonstrate only 5–13% relative to *S. anglica.* The test with NADP-ME indicated two *Zoysia* species expressed 30% and 42% relative to *Zea mays*; no detectable labelling with NADP-ME was shown for all other species studied. 

Thus, eleven species with only or predominant NAD-ME expression are defined as the NAD-ME biochemical subtype, and eight species (including *Zoysia*) with essential amounts of PEP-CK labelling were determined as the PEP-CK biochemical subtype (Table 1).

### 2.2. Light Microscopy

**Figure 2:**
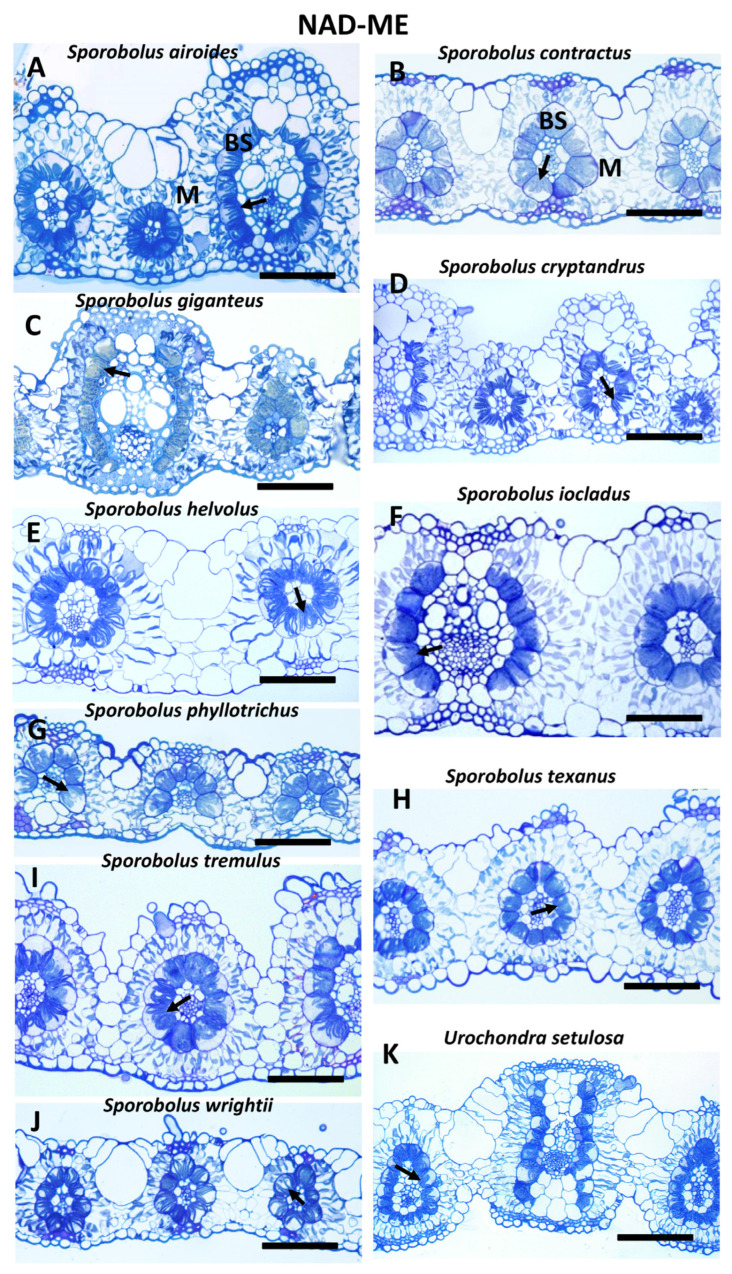
Light microscopy of leaf cross sections for 11 species of the tribe Zoysieae (Poaceae) that are classified as NAD-ME C_4_ biochemical subtype. Arrows indicate the centripetal (towards the vascular bundle) position of BS organelles. BS, bundle sheath; M, mesophyll. Scale: (**A**–**J**), 100 µm; (**K**), 200 µm.

**Figure 3:**
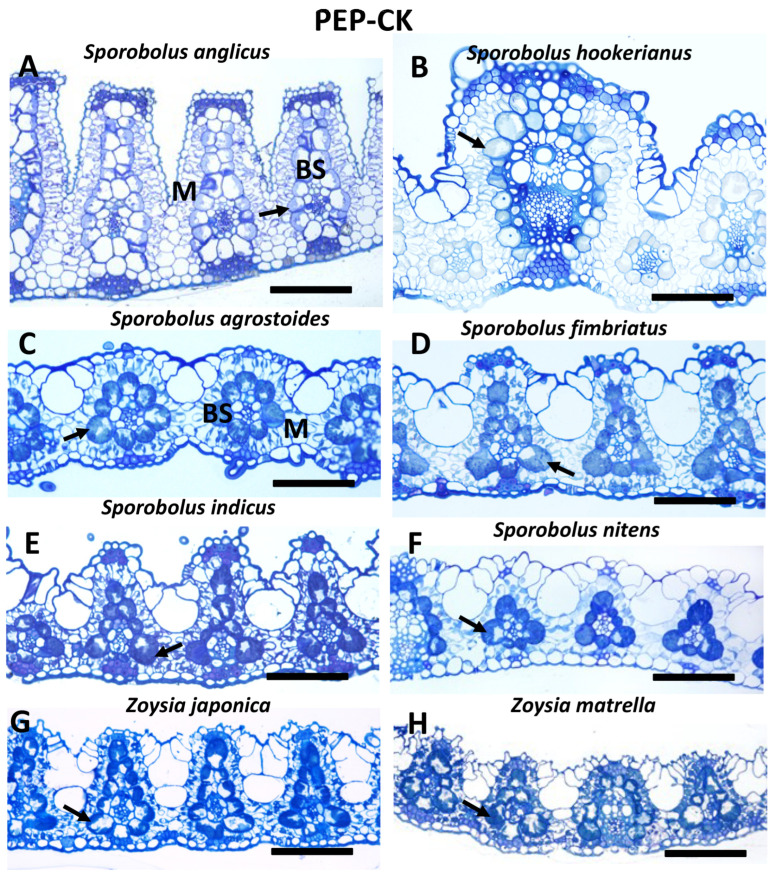
Light microscopy of leaf cross sections for eight species of the tribe Zoysieae (Poaceae) that are classified as PEP-CK C_4_ biochemical subtype. Arrows indicate centrifugal (towards the mesophyll) position of BS organelles. BS, bundle sheath; M, mesophyll. Scale: (**A**), 200 µm; (**B**–**H**), 100 µm.

The species studied differ in leaf morphology. Nine species have distinct ridges on the adaxial side, while on the abaxial side, the leaf surface is flat (Figure 2A,C,D,H,I and Figure 3A,B,D,E); in *U. setulosa,* both sides are undulated (Figure 2K), and in nine species, both abaxial and adaxial surfaces are nearly flat or slightly undulated (Figure 2B,E–G,J and Figure 3C,F–H). Leaf thickness measured across major veins differs more than three times between species from 312 µm in *S. anglicus* to about 90 µm in *Z. matrella* (Table 1). Minimum leaf thickness, as measured between veins, differs by about two times (Supplementary Table S2). In general, the highest diversity in leaf thickness was shown for NAD-ME species, while PEP-CK species mostly have thin leaves with two exceptions of former *Spartina* species (*S. anglicus* and *S. hookerianus*) (Figure 2 and Figure 3; Table 1). However, there is no significant difference (at *p* ≤ 0.05) in the average leaf thickness between NAD-ME and PEP-CK species. The thickest leaves are characteristic of species with ridges (except for *S. iocladus* and *S. helvolus*) (Figure 2 and Figure 3).

Species differ in vein architecture, having either three (midrib, majors and minors, *S. contractus*, *S. agrostoides*, *S. fimbriatus*, *S. giganteus*, *S. indicus*, *S. iocladus*, *S. phyllotrichus*, *S. texanus*, *S. tremulus*, *S. wrightii*, Zoysia), four (*S. airoides*, *S. anglicus*, *S. hookerianus*, *S. helvolus*, *S. nitens*), or five (*S. cryptandrus*) orders of veins; three species have the smallest veins as an additional under the bulliform cells (Figure 2A,D and Figure 3B). Interveinal distance (IVD) varies greatly among NAD-ME species, with a twofold difference between the smallest (about 100 µm in *S. cryptandrus*) and largest (about 200 µm in *S. iocladus* and *S. helvolus*). PEP-CK species in general have closer vein positioning, except for *S. anglicus* (Table 1). 

Kranz leaf anatomy in all studied species is characterized by two chlorenchymatous layers, outer mesophyll (M) cells and inner bundle sheath (BS) or Kranz cells surrounding vascular tissues (Figure 2 and Figure 3). In *S. phyllotrichus,* one to three BS cells on the adaxial side are not developed as chlorenchymatous; the BS in this case have an arc-like pattern (Figure 2G). Five of eight PEP-CK species have distinct BS extensions on the xylem pole of veins composed of BS cells, which do not have direct contact with vein tissues (Figure 3A,D,E,G,H). In these species, BS cells differ usually in size, with the biggest at the lateral abaxial side, which gives the veins a triangular outline (except for *S. anglicus and S. hookerianus*). The NAD-ME species *U. setulosa* also has specific BS extensions that are separated by empty parenchymatous cells (Figure 2K). The presence of a mestome sheath is characteristic for all species studied. However, in general, in minor veins it is not complete at the xylem pole, wherein the xylem vessels have direct contact with BS. Minor veins with a complete mestome sheath were found only in three species (*S. helvolus*, *S. iocladus*, *S. phyllotrichus*). All NAD-ME species studied have characteristic centripetal organelle positioning in BS cells (Figure 2; Supplementary Figure S2A,B), and the PEP-CK species studied have a centrifugal organelle distribution in BS cells (Figure 3; Supplementary Figure S2C,D).

### 2.3. Transmission Electron Microscopy

**Figure 4:**
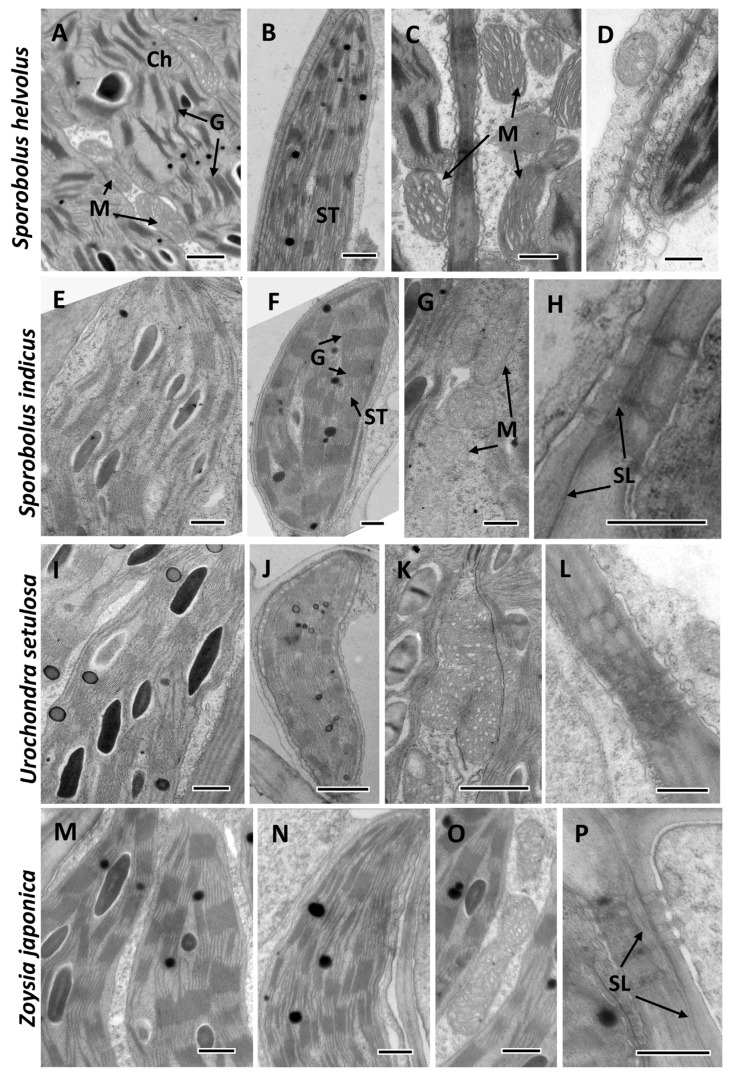
Ultrastructure of representative species of the tribe Zoysieae (Poaceae) that are classified as NAD-ME (**A**–**D**,**I**–**L**) and PEP-CK (**E**–**H**,**M**–**P**) C_4_ biochemical subtypes. (**A**,**E**,**I**,**M**) bundle sheath cell chloroplasts. (**B**,**F**,**J**,**N**) mesophyll chloroplasts. (**C**,**G**,**K**,**O**) bundle sheath cell mitochondria. (**D**,**H**,**L**,**P**) plasmodesmata pit field in the cell wall between BS and M, showing absence (**D**,**L**) and deposition (**H**,**P**) of suberin lamellae. G, grana; M, mitochondria; SL, suberin lamellae; ST, stromal thylakoid. Scale: (**A**–**C**,**J**–**K**) 1 µm; (**D**–**I**,**L**–**P**) 0.5 µm.

Analysis of the ultrastructural features showed characteristic differences in chloroplast structure for NAD-ME (Figure 4 illustrates *S. helvolus* and *U. setulosa)* and PEP-CK (Figure 4 illustrates *S. indicus* and *Z. japonica*) species. In NAD-ME species, BS chloroplasts have well-developed medium-sized grana (Figure 4A,I). The size and number of grana in M chloroplasts are smaller, but the main feature is the presence of numerous intergranal (stromal) thylakoids (Figure 4B,J). BS cells contain numerous mitochondria of variable sizes (Table 1 from the largest in *S. ioclados,* which are about 0.7 µm, to the smallest in *S. wrightii* and *S. texanus,* about 0.4 µm) that are located between chloroplasts in the centripetal position and have a specific tubular and/or lamellar cristae system (Figure 4C,K). Mitochondria are small and scarce in mesophyll (not shown). BS cell walls lack suberin lamellae (Figure 4D,L). In PEP-CK species, BS (Figure 4E,M) and M (Figure 4F,N) chloroplasts have a similar structure with a well-developed system of large sized grana (up to 20 or more thylakoids per grana). The mitochondria in PEP-CK species are located in the centrifugal position along with chloroplasts; their size, from the largest in *S. indicus,* which are about 0.7 µm, to the smallest in *Z. matrella* about 0.3 µm (Table 1), is comparable with those shown for NAD-ME species, but they are less numerous. Their tubular or crescent-like crista are less developed than in NAD-ME species (Figure 4G,O) but more developed compared to M. In all PEP-CK species, a suberin lamellae are recognized in the BS cell wall with characteristic thickening in the area of plasmodesmata pit fields between BS and M cells (Figure 4H,P).

### 2.4. Effect of Habitat MAP and MAT on Species Distribution and Anatomical Traits

**Figure 5:**
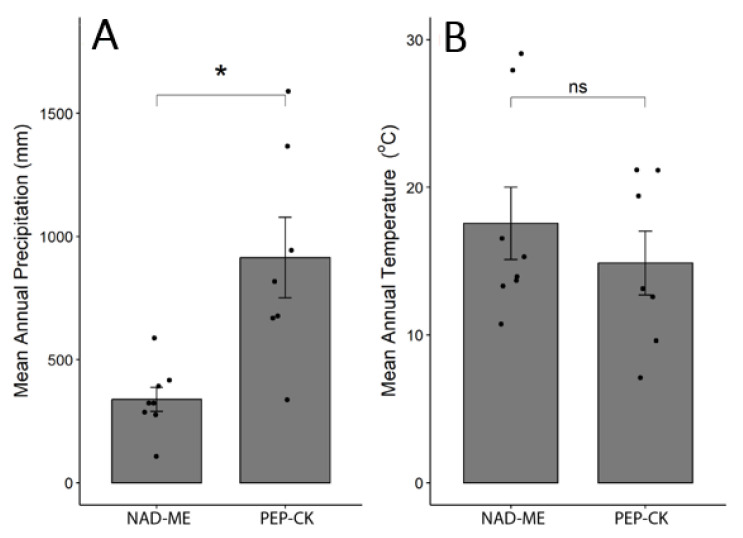
Habitat mean annual precipitation (**A**) and habitat mean annual temperature (**B**) for eight NAD-ME and seven PEP-CK C_4_ species used in current study. Gray bars indicate mean values ± SE (n = 7 and 8 species), and small black circles indicate the replicates. *t*-test results are indicated above the barplots, where ‘*’ indicates significant differences at *p* ≤ 0.05 and ‘ns’ indicates non-significant differences at *p* > 0.1.

An analysis of the relationship between mean annual precipitation (MAP) and distribution of species with different C_4_ biochemistry showed that in general, PEP-CK species preferred regions with higher MAP, while NAD-ME species were confined to arid (less than 300 mm of MAP) or semi-arid (300–550 mm of MAP, according to [18]) climates (Figure 5). Irrespective of C_4_ biochemical subtype, all species studied are distributed in regions with similar mean annual temperatures (MAT) (Figure 5). 

**Figure 6:**
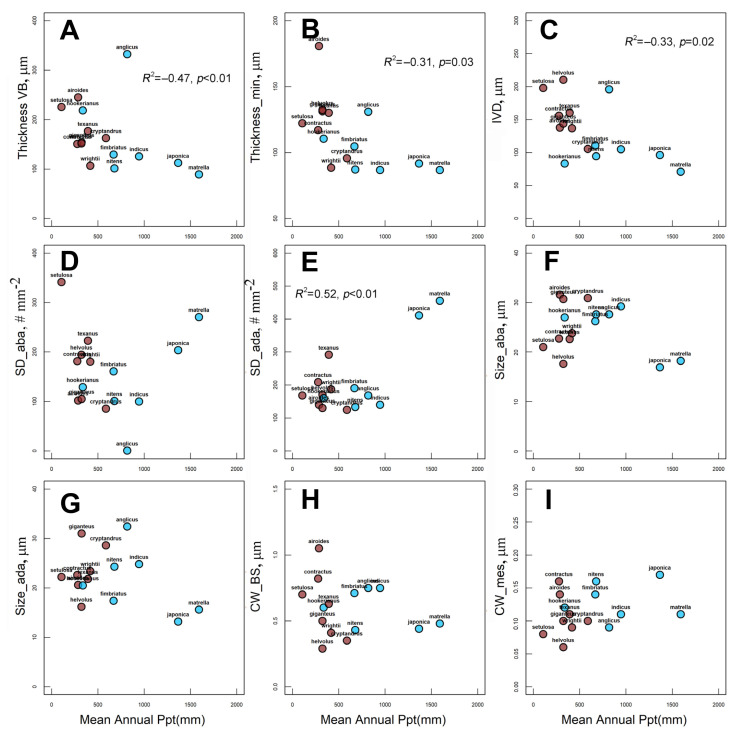
Relationship between mean annual precipitation (MAP) and leaf anatomical traits for 15 Zoysieae C_4_ grasses: (**A**) maximal (Thickness VB) and (**B**) minimal (Thickness_min) leaf thicknesses; (**C**) interveinal distance (IVD); (**D**) adaxial stomatal density (SD__ada_); (**E**) abaxial stomatal density (SD__aba_); (**F**) abaxial stomata size (Size_aba); (**G**) adaxial stomata size (Size_ada), µm; (**H**) BS (CW_BS) and (**I**) mesophyll (CW_mes) cell wall thicknesses. NAD-ME species are labeled with brown dots, PEP-CK species are labeled with blue dots. Regression coefficient (R^2^) is shown when *p* ≤ 0.03. For A and B, the regression coefficient was analyzed after exclusion of the influential point (*S. anglicus*).

**Figure 7:**
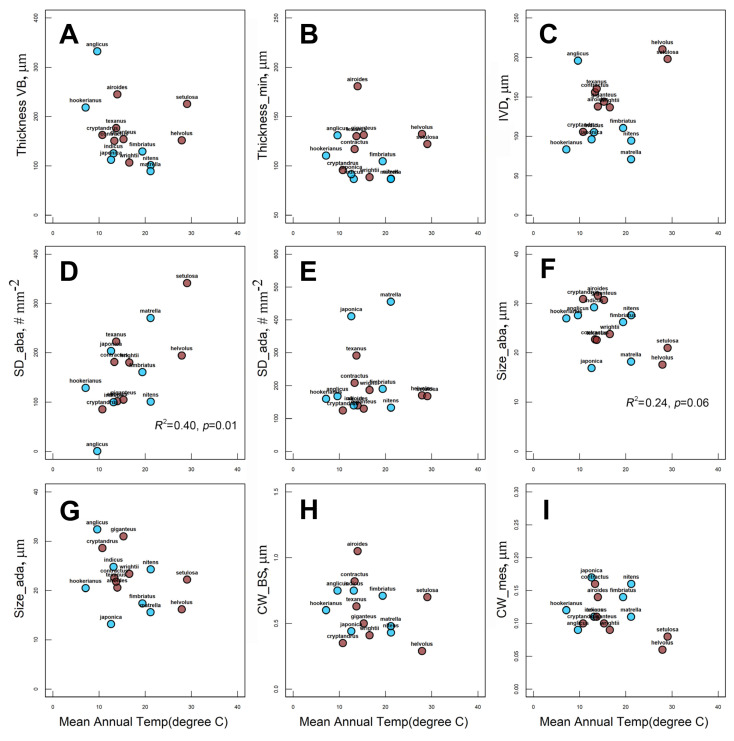
Relationship between mean annual temperature (MAT) and leaf anatomical traits for 15 Zoysieae C_4_ grasses: (**A**) maximal (Thickness VB) and (**B**) minimal (Thickness_min) leaf thicknesses; (**C**) interveinal distance (IVD); (**D**) adaxial stomatal density (SD__ada_); (**E**) abaxial stomatal density (SD__aba_); (**F**) abaxial stomata size (Size_aba); (**G**) adaxial stomata size (Size_ada); (**H**) BS (CW_BS) and (**I**) mesophyll (CW_mes) cell wall thicknesses. NAD-ME species are labeled with brown dots, PEP-CK species are labeled with blue dots. Regression coefficient (R^2^) is shown when *p* ≤ 0.06.

Values of leaf anatomical traits associated with photosynthesis (BS and M cell wall thickness, stomata number and sizes, leaf thickness and IVD) are shown in Table 1 and Supplementary Table S2. MAP showed a significant negative relationship only with IVD (Figure 6C). In addition, if the influential point, *S. anglicus,* is excluded from the analysis, there is a negative correlation between MAP and leaf thickness (maximal thickness, R^2^ = −0.47; *p* < 0.01 and minimal thickness, R^2^ = −0.31; *p* = 0.03) (Figure 6A,B). None of the other anatomical traits showed statistically significant relationships with MAP (Figure 6B,D–F). The significance shown for adaxial stomata density is affected by their high abundance in two *Zoysia* species, and is not confirmed after their exclusion. Among the several leaf structural traits studied, only abaxial stomatal density (R^2^ = 0.40, *p* = 0.008, Figure 7D) and abaxial stomatal sizes (R^2^ = −0.26, *p* = 0.06, Figure 7F) correlated with MAT. 

## 3. Discussion

### 3.1. Diversity in C_4_ Subtypes in Zoysieae

Among the 19 Zoysieae species in the current study, 10 *Sporobolus* species and *Urochondra setulosa* have NAD-ME as a predominant decarboxylation enzyme, with 5 of them characterized by additional expression of trace amounts of PEP-CK. Six *Sporobolus* and two *Zoysia* species have PEP-CK as a predominant decarboxylase, with NAD-ME as a complimentary decarboxylase. In this study, for 13 species, the biochemical subtype was recognized for the first time, while for 6 species, it was previously identified using decarboxylase enzyme activity measurements [4,5] or Western blotting [9,12] (see Supplementary Table S3 for the list of Zoysieae species studied, [36,37,38]). Leaf anatomical features in all Zoysieae species studied correspond to their biochemical subtypes. NAD-ME species are characterized by a centripetal distribution of chloroplasts in the bundle sheath (BS) cells and an absence of BS extensions. Chloroplasts have higher granal development in BS than in M, characteristic of this biochemical subtype. There are also numerous specific mitochondria in BS. All species of the PEP-CK subtype have a centrifugal positioning of chloroplasts in BS and BS extensions (except for *S. agrostoides*). BS cells have characteristic suberin depositions in cell walls. Chloroplasts in BS and M have nearly equally developed grana with extended stromal thylakoids. Mitochondria in PEP-CK species are numerous, and have relatively well-developed crista that can be associated with rather high levels of NAD-ME (Figure 1, Table 1), since NAD-ME is localized in mitochondria even in PEP-CK species, supporting ATP requirement for PEP-CK activity [9]. Combining the data obtained in this study with those published earlier, among the total 42 studied species of Zoysieae, 27 PEP-CK versus 15 NAD-ME C_4_ subtypes were recognized (Supplementary Table S3 with references). This accounts for about a fifth of the total number of species in Zoysieae, representing almost all sections of this tribe. 

Detection of NADP-ME enzyme protein in *Zoysia* in relatively high amounts (30–40% of maize) was surprising. Previously, the study of activity of three decarboxylases in *Zoysia japonica* showed very low values for NADP-ME (16–33 µmol mg chlorophyll h^−1^ versus 200–1000 in NADP-ME species), high values for the NAD-ME (63 µmol mg chlorophyll h^−1^ versus 120–600 in NAD-ME species), and high values for PEP-CK (but the lowest values among all PEP-CK species studied) [4]. It was mentioned that PEP-CK function was found in Chloridoideae and Panicoideae; however, only in the subfamily Panicoideae, PEP-CK appeared as additional in species with the NADP-ME subtype [36]. *Zea mays* is a well-known example from Panicoideae, containing NADP-ME and PEP-CK, which both act as CO_2_ delivery pathways via malate and aspartate [11,28]; however, this species is classified as having the NADP-ME biochemical subtype. The classical NADP-ME anatomical type with granal M and almost agranal BS chloroplasts confirms this classification in maize. Both the *Zoysia* species investigated in the current study have a classical PEP-CK chloroplast ultrastructure, with BS and M chloroplasts containing well-developed grana. This suggests that NADP-ME may make a limited contribution to the C_4_ cycle in *Zoysia*, which is possible if the enzyme activity is suppressed; a nonspecific immunoreaction of a protein with a molecular weight of 62 kDa is also possible.

### 3.2. Evolutionary Scenario of C_4_ Diversification

**Figure 8:**
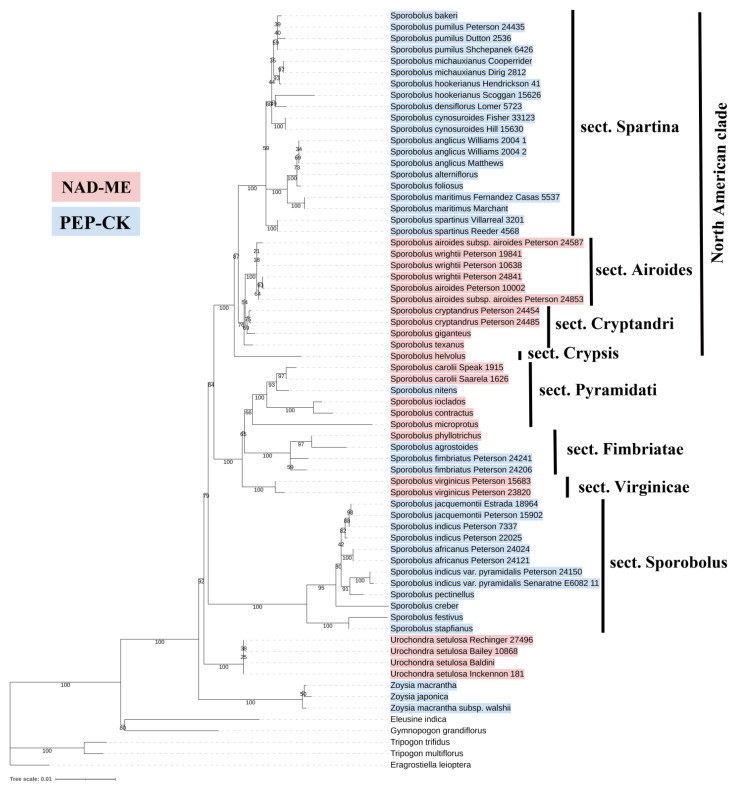
Molecular phylogenetic tree of tribe Zoysyeae based on combined plastome (rpl32-trnL, ndhA, rps16, and rps16-trnK) data for 35 species (67 accessions). Species with the NAD-ME C_4_ biochemical subtype are marked in red, and species with the PEP-CK C_4_ biochemical subtype are marked in blue.

To analyze C_4_ diversification, Zoysieae species with a known C_4_ biochemical subtype were positioned on a phylogenetic tree inferred from combined plastid (rpl32-trnL, ndhA, rps16, and rps16-trnK) sequences (Figure 8). For this analysis, along with species with that biochemical subtype confirmed via appropriate methods, species classified according to anatomy were included (for the list of species, see Supplementary Table S3). We considered this possible because to date, the anatomical types of the studied Zoysieae species correspond to biochemical subtypes. The sections on the tree were recognized according to [33]. The results show heterogeneity in C_4_ biochemical subtypes in Zoysieae, which indicates multiple switches between PEP-CK and NAD-ME photosynthetic subtypes, especially within *Sporobolus* species. Early diverged *Sporobolus* species have a Sporobolus section and contain exclusively PEP-CK species. The most heterogeneous biochemical composition is confined to sections Pyramidati and Fimbriatae. The North American clade consists of several sections, and almost all of them are NAD-ME, except for the Spartina clade that is PEP-CK (Figure 8). Genus *Spartina* only recently was incorporated into *Sporobolus* based on molecular phylogenetic data [34]; however, it has some anatomical features related to adaptation to saline environments [25,39] that distinguish it from other *Sporobolus*. 

It is difficult to reveal the direction of evolutionary transitions without special genetic and phenotypic analyses [40]. Discussion of C_4_ subtype diversification depends on the ancestral type reconstruction, but it usually would not have been detectable via a phylogenetic tree of species [40]. It is suggested that among the 22–24 independent origins of C_4_ cycle in grasses, the subfamily Chloridoideae represents one event [20,21]. Phylogenetic analyses on *pck* sequences suggest the C_4_ PEP-CK subtype seems to have evolved during the early diversification of the subfamily Chloridoideae, followed by several switches between PEP-CK and NAD-ME [36]. According to this scenario, there are four distinct C_4_-*pck* gene lineages, *pck*-B, -C, -D, -E. Among the 57 grass species sampled, 4 Zoysieae representatives were used in analyses; in *Sporobolus africanus* and *S. festivus* (both from section Sporobolus), the ancestral *pck*-B is detected, while in *Zoysia japonica* and *Spartina maritima,* the PEP-CK function was secondarily acquired with changes in the *pck* gene [36]. Thus, based on analysis of our and previous data, the PEP-CK subtype could be inferred as ancestral for the tribe Zoysieae, with *Zoysia* (PEP-CK) positioned as a basal clade, or for the genus *Sporobolus,* with section Sporobolus (all species are PEP-CK) as a basal clade. All NAD-ME species in Zoysieae represent multiple PEP-CK function losses: a single loss in *Urochondra,* a single loss in a common ancestor of the North American clade, and multiple (at least two) events in *Sporobolus* sections Fimbriatae and Piramidati. Some trace amounts of PEP-CK in NAD-ME species, which is quite a rare event among NAD-ME species [12], may be a residual effect of this loss. The position of former *Spartina* species within NAD-ME clades on phylogenetic tree demonstrates secondary reacquisition of PEP-CK that is in accordance with *pck*-phylogeny [36]. In [36], *Zoysia* species were also treated as an independent reversion to PEP-CK, which is not clear from the species’ position as a basal clade (Figure 8). Anatomically *Zoysia* species are similar to PEP-CK *Sporobolus* (except for the former *Spartina*), but the expression of the third decarboxylase, NADP-ME, distinguishes them from others. 

In the current study, PEP-CK species show the different molecular weights of PEP-CK on immunoblots: *Sporobolus*—69 kDa, *Zoysia*—74 kDa, former *Spartina*—double bands 69 + 74 kDa. This difference corresponds to three different lineages in Zoysieae according to *pck* molecular phylogeny [36], and could be related to genetic changes in *pck*. Variability in the molecular mass of PEP-CK previously was illustrated with smaller mass in *Sporobolus* species (69 kDa, *S. pyramidalis*, *S. indicus* and *S. jacquemontii*), larger mass (71 kDa) in *S. anglicus* (*Spartina anglica*) and *Panicum maximum* [28], and the largest mass (74 kDa) in maize. Moreover, PEP-CK in *Sporobolus* was not phosphorylated in the dark, in contrast to *Panicum* species [28]. Besides, *Spartina* shows double lines [12] that could be related to the rates of proteolytic cleavage [41]. 

### 3.3. Habitat Effect on Zoysieae Diversification

It is well known that C_4_ photosynthesis is advantageous under conditions in which CO_2_ becomes a limiting factor, mainly high temperature and drought, and the distribution pattern of C_4_ subtypes depends on climate [18,42,43,44]. As was shown for grasslands of Australia, Namibia, South Africa, Israel, and United States, among grasses, NAD-ME species are predominantly found in drier regions; however, NADP-ME species prefer habitats with increased annual precipitation [18,45,46,47]. Data on the environmental preferences of the PEP-CK subtype are inconsistent because PEP-CK species are less common, and that specific subtype can be mistyped based on leaf anatomy [18]. With the increase in annual rainfall, the number of PEP-CK species has increased in Australia [18] and Israel [47], decreased in the US [46], and has not shown a clear tendency in Argentina [45]. Unlike the precipitation effect, the temperature preferences of different subtypes have been less frequently analyzed; no significant dependence has been revealed [45]. 

Considering the multiple transitions in biochemistry and anatomy among phylogenetically closely related species, we further assessed the ecological impact of habitat, especially temperature and water availability, on the species distribution. In contrast to previous studies wherein the few representative species of separate lineages were used for biogeographical distribution [48], in our study, comparison was carried out within phylogenetically related species that grow on the different continents [33,34]. Our results show a strong correlation between two C_4_ biochemical subtypes and annual precipitation, with NAD-ME Zoysieae species confined to drier habitats and PEP-CK species preferring wet areas. At the same time, there is no evidence of temperature dependency in species distribution in Zoysieae. Our results are consistent with previous data on different grassland analyses [18,47], and are in accordance with the greater drought resistance of NAD-ME over PEP-CK representatives of Chloridoideae [49,50]. This suggests that multiple losses of PEP-CK decarboxylase could have been driven by climate aridization. 

There is still limited understanding of what physiological or anatomical traits are responsible for the different adaptive capacity of C_4_ biochemical subtypes. Our analyses show species from drier habitats have thicker leaves, wherein the higher thickness across VB is associated in many species with distinct leaf ridges (with the maximal ridge height in *S. anglicus*). Additionally, increased leaf thickness is correlated with higher IVD. These traits allow for a reduction in leaf conductance and water loss by increasing the boundary layer path from veins and by hiding stomata deep in furrows on the adaxial leaf side, or even on both (e.g., in *Urochondra*) leaf sides. Previously, an analysis of 18 C_4_ grasses from various PACMAD lineages showed that thicker leaves and greater IVD under lower MAP conditions correlated with greater mesophyll conductance and low leaf hydraulic conductance, resulting in high rates of photosynthesis and water use efficiency [51]. Apparently, the higher heterogeneity in these traits in NAD-ME plants is associated with an adaptation gradient from arid to semi-arid climates. With a wider range of humidity in PEP-CK plant habitats (from moderate to high MAP), variation in anatomical parameters is much lower. Thus, this leaf-level trait variability is related mostly to environmental adaptations, rather than to biochemical subtype. 

Modifications in leaf anatomy are often recognized as associated with specific adaptations to support metabolism under stressful habitat conditions. Cell wall structure, including thickness, affects mesophyll and hydraulic conductance in C_3_ species [52,53,54], thereby limiting photosynthetic and water use efficiency. Our results for C_4_ species show no correlation of CW thickness with either environmental factors or C_4_ biochemical subtype, suggesting a species specificity for this trait. Suberin deposition in BS CW reduces CO_2_ leakage across the BS–M interface during C_4_ cycle in species with NADP-ME and PEP-CK decarboxylation [55]. In Zoysieae, all PEP-CK species have a characteristic suberin lamella in BS CW; at the same time, the BS CW thickness varies, and does not depend on habitat conditions and biochemical subtype. Higher adaxial stomatal density and smaller stomata size were found to be positively affect photosynthetic efficiency, creating additional parallel pathways for CO_2_ diffusion in C_4_ plants that is beneficial for the open-area habitat [56]. However, our analysis shows adaxial stomata are not affected by habitat MAP and MAT, while abaxial stomata increase in number and decrease in size under higher MAT. In grasses, selective reactions of stomata pattering on either leaf side depend on leaf exposition, ability to curve, specific habitat adaptation, and grass lineage [25,57,58], which need to be tested additionally for closely related C_4_ grasses. This suggests stomata traits respond differently depending on plant lineage and habitat, consistent with the fact that no common adaptive response of stomata to changing conditions has been identified among plants.

## 4. Materials and Methods

### 4.1. Plant Growth Conditions

The nineteen C_4_ grasses (listed in Supplementary Table S1 together with the seed source) belong to three genera (*Sporobolus*, *Zoysia*, and *Urochondra*) from tribe Zoysieae, subfamily Chloridoideae, family Poaceae. Plants were raised from seeds except for two *Zoysia* species received from USDA as living plants. Plants were grown in the Washington State University (Pullman, WA, USA) greenhouse during the mid-winter/spring months, with day/night temperatures ~26/18 °C and a maximum mid-day PPFD of 1000 µmol photosynthetic quanta m^−2^ s^−1^. For each species, three individual plants were grown in two L pots with commercial potting soil (a Sunshine Mix LC-1 soil, Sun Gro Horticulture, Agawam, MA, USA). They were watered daily, or every other day, and nutrients were provided once per week via watering with Scotts Peter’s Professional fertilizer (N-P-K 20:20:20; Scotts Miracle-Gro, Marysville, OH, USA) at a concentration of 500 ppm for each element. After three months of growth, samples of mature fully expanded leaves were taken from each plant for microscopy and biochemical analyses. The plant growth and sample collection were repeated two times. 

### 4.2. Light and Scan and Transmission Electron Microscopy

To examine the type of leaf anatomy, the mid portion of fully expanded leaves was sampled for all species studied. They were fixed at 4 °C in 2% (*v*/*v*) paraformaldehyde and 2% (*v*/*v*) glutaraldehyde in 0.1 M phosphate buffer (pH 7.2), post-fixed in 2% (*w*/*v*) OsO_4_, and, after an acetone dehydration procedure, embedded in Spurr’s epoxy resin. Leaf cross-sections (1 µm thick), which were made using a Leica EM UC6 ultramicrotome (Leica Microsystems, Wetzlar, Germany), were stained with 1% (*w*/*v*) toluidine blue O in 1% (*w*/*v*) Na_2_B_4_O_7_ and observed with the AxioScope A1 (Zeiss, Jena, Germany) light microscope. Light microscopy images of leaf cross-sections were used to measure leaf thickness across (Thickness_VB) and between (Thickness_min) vascular bundles, and interveinal distance (IVD) was measured using an image analysis program (ImageJ 1.37v, Wayne Rasband, National Institutes of Health, Stapleton, NY, USA). 

For electron microscopy, ultra-thin sections (70 nm thick) were stained with 2% (*w*/*v*) uranyl acetate, followed by 2% (*w*/*v*) lead citrate. A Zeiss Libra 120 transmission electron microscope (Oberkochen, Germany) was used for observation and photography. Bundle sheath and mesophyll cell wall thicknesses (CW_BS, CW_M) were measured from transmission electron microscope micrographs, using at least 20 images for each species.

To calculate the stomatal number and to measure their sizes, the adaxial and abaxial epidermal surfaces were captured on three leaves per species in low-vacuum mode with a FEI Scanning Electron Microscope Quanta 200F (FEI Co., Hillsboro, OR, USA, Field Emission Instruments). The number of stomata was expressed per mm^2^ as adaxial stomatal density (SD_ada_) and abaxial stomatal density (SD_aba_). 

### 4.3. Western Blot Analysis

Total proteins were extracted separately from leaves of two or three individual plants by homogenizing 0.2 g of tissue in 0.4 mL of extraction buffer (100 mM Tris-HCl, pH 7.5, 10 mM (*w*/*v*) MgCl_2_, 1 mM (*w*/*v*) EDTA, 15 mM (*v*/*v*) β-mercaptoethanol, 20% (*v*/*v*) glycerol, and 1 mM phenylmethylsulfonyl fluoride). Extraction was continued by adding 0.4 mL 60 mM Tris-HCl, pH 7.5, 4% (*w*/*v*) SDS, 20% (*v*/*v*) glycerol, 0.5% (*v*/*v*) β-mercaptoethanol, and 0.1% (*w*/*v*) bromphenol blue. After boiling for 5 min for SDS-PAGE, the supernatant was collected after centrifugation at 14,000× *g* for 5 min, and protein concentration was determined with an RCDC protein quantification kit (Bio-Rad, Hercules, CA, USA). Protein samples (20 µg) were separated by 10% SDS-PAGE, blotted onto nitrocellulose, stained with Ponceau S for a loading control, and probed with anti-*Amaranthus hypochondriacus* NAD-ME IgG that was prepared against the 65 KDa α subunit, courtesy of J. Berry [59] (1:2000); anti-*Zea mays* 62 kDa NADP-ME IgG, courtesy of C. Andreo [60] (1:2500); and anti-*Urochloa maxima* PEP-CK IgG, courtesy of R. Walker (Università degli Studi di Perugia, Italy) (1:5000); and treated overnight at 4 °C. Goat anti-rabbit IgG-alkaline phosphatase conjugated secondary antibodies (Sigma, Kawasaki, Kanagawa, Japan) were used at a dilution of 1:10,000 for detection. Bound antibodies were visualized by developing the blots with 20 mM nitroblue tetrazolium and 75 mM 5-bromo-4-chloro-3-indolyl phosphate in detection buffer [100 mM Tris-HCl, pH 9.5, 100 mM NaCl, and 5 mM MgCl_2_]. A minimum of two separate blots from two separate extractions were made for each enzyme. Species were grouped to fit within 15 gel lines, including reference species and/or protein molecular weight marker, and were regrouped for a second or third replicate. There are 12 blots in total. To compare the relative amounts of decarboxylases in all species, the reference protein samples were added to each set of species: *S. anglicus* for PEP-CK, *S. airoides* for NAD-ME, and *Zea mays* for NADP-ME (see Supplementary Figure S1 for the representative blots). The intensities of bands in Western blots were quantified with an image analysis program (ImageJ 1.37v), and expressed relative to mean level in *S. anglicus* for PEP-CK, in *S. airoides* for NAD-ME, and in *Zea mays* for NADP-ME, which was set at 100%. 

### 4.4. Phylogenetic Tree Reconstruction

For phylogeny reconstruction, four chloroplast markers were used: *rpl32-trnL*, *ndhA*, *rps16*, *rps16-trnK* (for accessions, see Supplementary Sporobolus_accession.xlsx). The data for each voucher were concatenated, and then alignment was created using mafft v7.310 [61] (parameters: –maxiterate 1000, –globalpair) followed by minor manual editing in MEGA X [62]. The phylogenetic analysis was conducted in IQ-TREE2 v2.2.0 [63] using the maximum likelihood method, with 1000 bootstrap replicates and a substitution model estimation for each gene (parameters: −p, −b 1000). The majority-rule consensus tree was used as the resulting tree. In total 72 plastome sequences, data were retrieved from NCBI, including 67 accessions of Zoysieae species (35 species); 4 species selected as the outgroup (from tribes Cynodonteae, Chloridoideae). 

### 4.5. Habitat Mean Annual Precipitation and Mean Annual Temperature

The global distribution data for the geo-referenced species were taken from the Global Biodiversity Information Facility (GBIF; http://www.gbif.org/ (accessed on 19 November 2023)) site using the gbif function in R package (version 3.5.2) dismo [64]. Values for mean annual temperature (MAT) and mean annual precipitation (MAP) for the period from 1970 to 2000 for all geo-referenced localities for each species were extracted from the WorldClim dataset (http://www.worldclim.org/ (accessed on 19 November 2023)) using the extract function in R package raster [64]. The values were then averaged as the MAT and MAP value for a given species. The data on MAP and MAT were only available for 15 species (see Supplementary Table S1 for the number of records). 

### 4.6. Statistical Analysis

Statistical analyses were performed using R software (version 3.5.2; R Foundation for Statistical Computing, Vienna, Austria). Regression analyses were performed, using the mean values of traits for each of the 19 species, in order to examine the relationships of leaf anatomical traits with habitat MAP and MAT. For regression analyses, values of *p* ≤ 0.05 were considered statistically significant, and those of *p* ≤ 0.1 were considered as marginally significant. A one-way analysis of variance (ANOVA) with post hoc Tukey’s test was used to examine differences in leaf-level anatomical and biochemical traits among the 19 Zoysieae grasses. For the one-way ANOVA, values of *p* ≤ 0.05 were statistically significant. 

## 5. Conclusions

Sufficient sampling is crucial for revealing the evolution of C_4_ photosynthetic subtypes in the separate phylogenetic lineages. Containing greatest number of C_4_ species, the family Poaceae has the smallest proportion of species with a clearly defined biochemical subtype. Tribe Zoysieae is entirely composed of C_4_ species, and here we proposed a scenario of the diversification of C_4_ biochemical and anatomical subtypes using data for 42 species, including the 19 species analyzed in current paper. The phylogenetic pattern of C_4_ biochemical subtypes indicates multiple transitions between NAD-ME and PEP-CK function, with confirmed events of PEP-CK reacquisition. This finding shows that reversions within of C_4_ photosynthetic type occur more frequently than reversions between C_3_ and C_4_ [40]. 

Analysis of Zoysieae habitats allowed us to reveal a strong correlation between C_4_ biochemical subtype and MAP, where NAD-ME Zoysieae species are confined to drier habitats and PEP-CK species prefer humid areas. Thus, we provided comparative statistical evidence that climate aridization could be treated as a trigger for the loss of PEP-CK function during the diversification of Zoysieae species. Further anatomical adjustments enable plants to optimize photosynthesis during acclimation to drought in their ecological niches. Altogether, we would like to highlight the potential for investigation of C_4_ evolution in separate grass lineages characterized by multiple events of diversification, including loss and reversion of the primary biochemical subtype followed by anatomical optimization.

## Figures and Tables

**Table 1:** Quantitative representation of Western blot data for three decarboxylases, PEP-CK, NAD-ME, and NADP-ME; classification of species according to their biochemical subtype, and leaf anatomical traits related to photosynthesis. Different letters indicate significant differences between species in a column at *p* ≤ 0.05. BS, bundle sheath; cf, centrifugal; cp, centripetal; CW, cell wall; M, mesophyll.

	Band Intensity in % to Reference Species *			Biochemical Subtype	BS Chloroplast Position	BS CW Suberin	Leaf Thickness **, µm	Interveinal Distance, µm	BS Mitochondria Size, Short Axes, µm
	PEP-CK	NAD-ME	NADP-ME						
*S. airoides*	7 ± 3.3 d	100 b	0	NAD-ME	cp	-	245.20 ± 3.23 b	137.86 ± 2.57 c	0.61 ± 0.02 bc
*S. contractus*	0	96 ± 6.9 b	0	NAD-ME	cp	-	150.95 ± 1.87 d	156.00 ± 2.79 bc	0.59 ± 0.03 bc
*S. cryptandrus*	3 ± 2.3 d	93 ± 5.9 b	0	NAD-ME	cp	-	162.58 ± 6.29 cd	105.70 ± 2.83 d	0.59 ± 0.03 bc
*S. giganteus*	0	90 ± 7.5 bc	0	NAD-ME	cp	-	154.4 ± 3.24 d	143.9 ± 3.08 c	0.56 ± 0.03 c
*S. helvolus*	13 ± 2.3 d	75 ± 6.0 c	0	NAD-ME	cp	-	151.92 ± 8.04 cd	210.43 ± 7.13 a	0.68 ± 0.05 ab
*S. ioclados*	5 ± 3.2 d	137 ± 33.4 ab	0	NAD-ME	cp	-	222.41 ± 5.00 b	213.08 ± 9.71 a	0.71 ± 0.03 ab
*S. phyllotrichus*	0	122 ± 8.6 a	0	NAD-ME	cp	-	101.54 ± 5.55 fg	135.97 ± 3.07 c	0.50 ± 0.03 c
*S. texanus*	0	138 ± 7.3 a	0	NAD-ME	cp	-	176.93 ± 6.42 c	160.39 ± 6.11 b	0.42 ± 0.02 d
*S. tremulus*	0	91 ± 13.8 bc	0	NAD-ME	cp	-	173.50 ± 3.97 c	165.55 ± 4.15 b	0.82 ± 0.04 a
*S. wrightii*	0	90 ± 7.2 bc	0	NAD-ME	cp	-	106.85 ± 3.70 f	136.94 ± 4.51 c	0.38 ± 0.01 d
*U. setulosa*	10 ± 3.5 d	73 ± 2.6 c	0	NAD-ME	cp	-	225.60 ± 9.54 b	197.97 ± 4.01 a	0.69 ± 0.03 b
Average for NAD-ME species	3.4 ***	100.5 ***	0 ***				170.2 ns	160.3 ***	0.60 ns
*S. anglicus*	100 b	49 ± 9.3 cd	0	PEP-CK	cf	+	312.38 ± 14.71 a	182.73 ± 7.55 a	0.66 ± 0.06 b
*S. agrostoides*	59 ± 3.23 c	53 ± 8.1 cd	0	PEP-CK	cf	+	124.82 ± 2.91 e	127.40 ± 3.61 c	0.41 ± 0.02 d
*S. fimbriatus*	58 ± 3.2 c	24 ± 5.3 e	0	PEP-CK	cf	+	129.36 ± 3.87 e	110.56 ± 3.69 d	0.41 ± 0.02 d
*S. hookerianus*	111 ± 13.2 b	59 ± 9.4 cd	0	PEP-CK	cf	+	223.20 ± 6.33 b	83.44 ± 1.80 e	0.65 ± 0.05 b
*S. indicus*	67 ± 5.5 c	33 ± 10.2 de	0	PEP-CK	cf	+	125.78 ± 3.44 e	105.16 ± 3.22 d	0.45 ± 0.01 d
*S. nitens*	61 ± 6.4 c	25 ± 5.3 e	0	PEP-CK	cf	+	101.58 ± 4.14 fg	94.57 ± 4.25 d	0.53 ± 0.02 cd
*Z. japonica*	196 ± 23.6 a	94 ± 15.7 ab	42 ± 5.7 a	PEP-CK	cf	+	112.44 ± 2.89 ef	96.24 ± 2.06 d	0.42 ± 0.01 d
*Z. matrella*	155 ± 31.2 a	73 ± 2.3 c	30 ± 4.1 a	PEP-CK	cf	+	89.20 ± 2.32 g	71.02 ± 1.32 f	0.30 ± 0.01 e
Average for PEP-CK species	100.9	51.3	36				163.8ns	140.4	0.43

* The reference species for NAD-ME is *S. airoides*, for NADP-ME is *Zea mays*, and for PEP-CK is *S. anglicus*. They were set as 100% at corresponding blots. ** Leaf thickness measured across vascular bundle on leaf section. *** Significant differences between NAD-ME and PEP-CK groups at *p* ≤ 0.05. ns indicates non-significant differences between NAD-ME and PEP-CK groups at *p* > 0.1.

## Data Availability

All data supporting the findings of this study are available within the paper and within its Supplementary Materials published online.

## Supplementary Materials

The following supporting information can be downloaded at: https://www.mdpi.com/article/10.3390/plants12234049/s1, Figure S1. Western blots for three decarboxylases from soluble proteins extracted from leaves of grass species, and representative membranes stained with Ponceau S after protein transfer to nitrocellulose membrane and before immunoblotting. Figure S2. Light and electron microscopy images of leaf cross-sections showing organelle positioning in grasses of different biochemical subtypes: A, B. *Sporobolus texanus*, NAD-ME species with centripetal position (towards the vascular bundle) of organelles in BS cells; *Sporobolus agrostoides*, PEP-CK species with centrifugal position (towards the mesophyll cells) of organelles in BS cells. Table S1. List of Zoysieae species studied, source of seeds, and GBIF records. Table S2. Leaf anatomical traits related to photosynthesis. Table S3. List of Zoysieae species with suggested C_4_ biochemical subtype based on different methods with references. Table S4. *Sporobolus* accessions used for phylogeny reconstruction.
