# Supplementary material for: Biochemical and Structural Diversification of C4 Photosynthesis in Tribe Zoysieae (Poaceae)
Source: Plants (Basel). 2023 Nov 30;12(23):4049. doi: 10.3390/plants12234049 (PMC10798372; doi:10.3390/plants12234049)
Supplement: Supplementary file 1 [file plants-12-04049-s001.zip › plants-2699636-supplementary.pdf]

Supplementary file

Biochemical and Structural Diversification of C_4 Photosynthesis in Tribe Zoysieae (Poaceae)

Nuria K. Koteyeva [1,*], Elena V. Voznesenskaya [1,*], Varsha S. Pathare [2], Tatyana A. Borisenko [1,3], Peter M. Zhurbenko [4], Grigory A. Morozov [5] and Gerald E. Edwards [6]

[1] Laboratory of Anatomy and Morphology, Komarov Botanical Institute of Russian Academy of Sciences, 197376 St. Petersburg, Russia.

[2] Institute for Genomic Biology, University of Illinois at Urbana-Champaign, Champaign, IL 61801, USA; varsha.pathare@wsu.edu

[3] Faculty of Biology, St. Petersburg State University, 199034 St. Petersburg, Russia; st075629@student.spbu.ru

[4] Laboratory of Biosystematics and Cytology, Komarov Botanical Institute of Russian Academy of Sciences, 197376 St. Petersburg, Russia; pj_28@mail.ru

[5] Chair of Medical Biology, North-Western State Medical University named after I.I. Mechnikov, 191015 St. Petersburg, Russia; dartost1@rambler.ru

[6] School of Biological Sciences, Washington State University, Pullman, WA 99164-4236, USA; edwardsg@wsu.edu

Supplementary Figure S1. A. Representative Western blots for three decarboxylases from total proteins extracted from leaves of Zoysieae grass species, and representative membrane stained with Ponceau S after proteins (20 µg per lane) transfer to nitrocellulose membrane and before immunoblotting. B. Additional representative blots to show all species used in study. Blots were probed with antibodies raised against PEP-CK, NAD-ME, and NADP-ME. Reference species for NAD-ME is *S. airoides*, for NADP-ME is *Zea mays*, for PEP-CK is *S. anglicus*. The molecular mass is indicated to the sides of the blots. Where indicated, grass species are marked that are not included in the current paper, but they are retained to avoid modifying the blots.

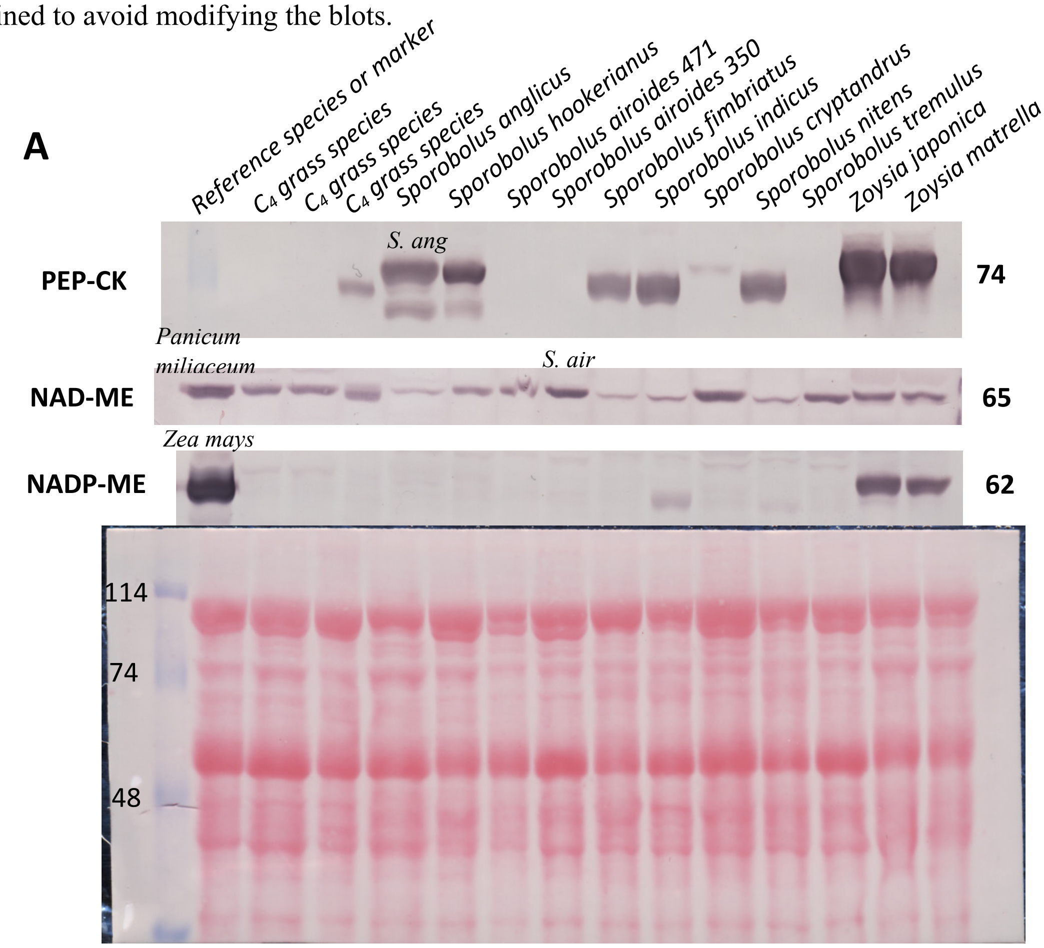

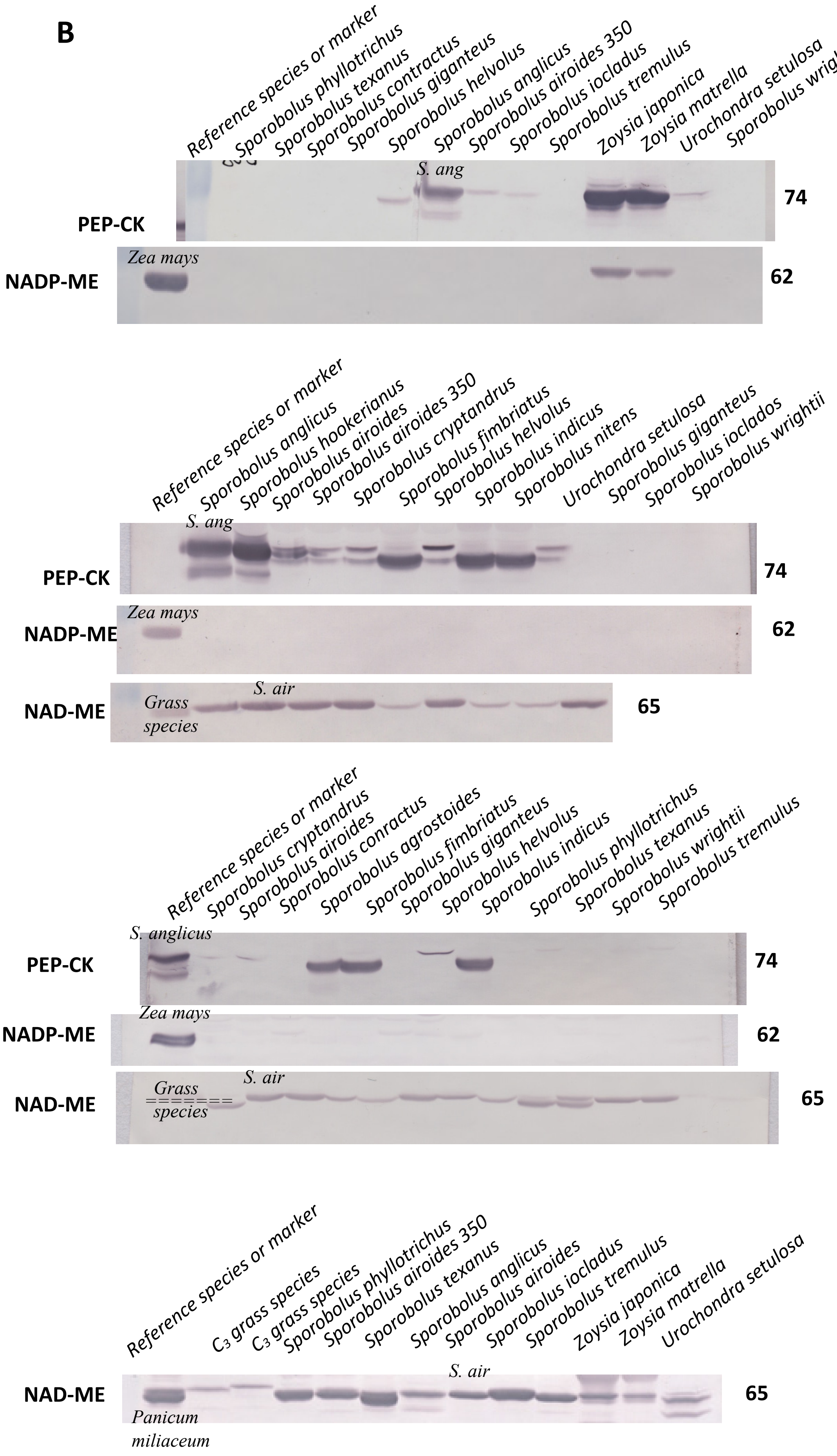
B
Reference species or marker
Sporobolus phyllotrichus
Sporobolus texanus
Sporobolus contractus
Sporobolus giganteus
Sporobolus helvolus
Sporobolus anglicus
Sporobolus airoides 350
Sporobolus iocladus
Sporobolus tremulus
Zoysia japonica
Zoysia matrella
Urochondra setulosa
Sporobolus wrightii
S. ang
PEP-CK
74
Zea mays
NADP-ME
62
Reference species or marker
Sporobolus anglicus
Sporobolus hookerianus
Sporobolus airoides
Sporobolus airoides 350
Sporobolus cryptandrus
Sporobolus fimbriatus
Sporobolus helvolus
Sporobolus indicus
Sporobolus nitens
Urochondra setulosa
Sporobolus giganteus
Sporobolus ioclados
Sporobolus wrightii
S. ang
PEP-CK
74
Zea mays
NADP-ME
62
Grass species
S. air
NAD-ME
65
Reference species or marker
Sporobolus cryptandrus
Sporobolus airoides
Sporobolus conractus
Sporobolus agrostoides
Sporobolus fimbriatus
Sporobolus giganteus
Sporobolus helvolus
Sporobolus indicus
Sporobolus phyllotrichus
Sporobolus texanus
Sporobolus wrightii
Sporobolus tremulus
S. anglicus
PEP-CK
74
Zea mays
NADP-ME
62
Grass species
S. air
NAD-ME
65
Reference species or marker
C3 grass species
C3 grass species
Sporobolus phyllotrichus
Sporobolus airoides 350
Sporobolus texanus
Sporobolus anglicus
Sporobolus airoides
Sporobolus iocladus
Sporobolus tremulus
Zoysia japonica
Zoysia matrella
Urochondra setulosa
S. air
NAD-ME
65
Panicum miliaceum

Supplementary Figure S2. Light and electron microscopy images of leaf cross-sections showing organelle positioning in grasses of different biochemical subtypes: A, B. *Sporobolus texanus*, NAD-ME species with centripetal position (towards the vascular bundle) of organelles in BS cells; *Sporobolus agrostoides*, PEP-CK species with centrifugal position (towards the mesophyll cells) of organelles in BS cells. BS, bundle sheath; Ch, chloroplast; M, mesophyll cells; V, vacuole; VB, vascular bundle. Scales: A, C, 50 µm; B, D, 10 µm.

Sporobolus texanus

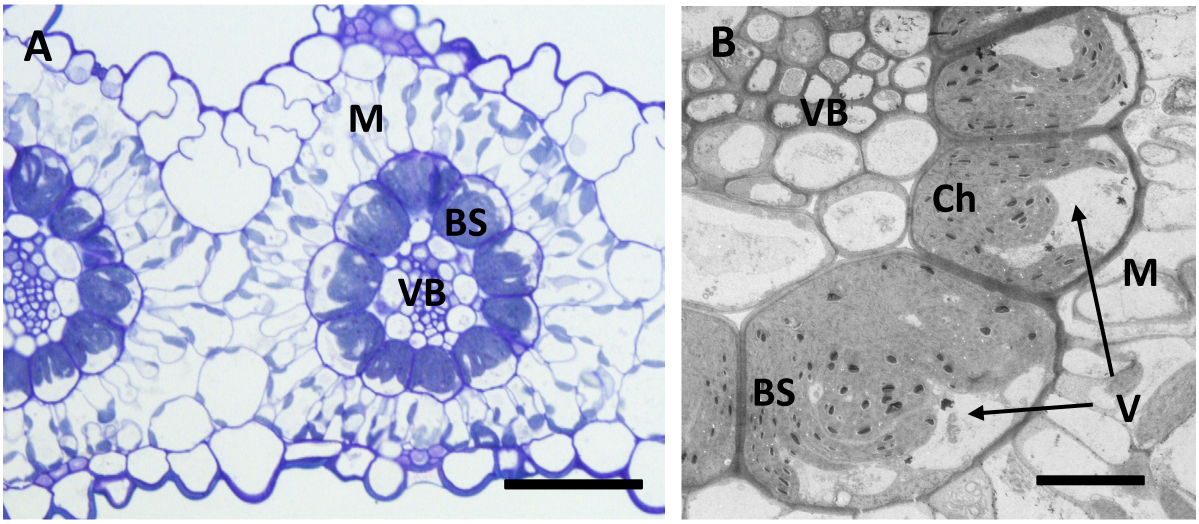

Sporobolus agrostoides

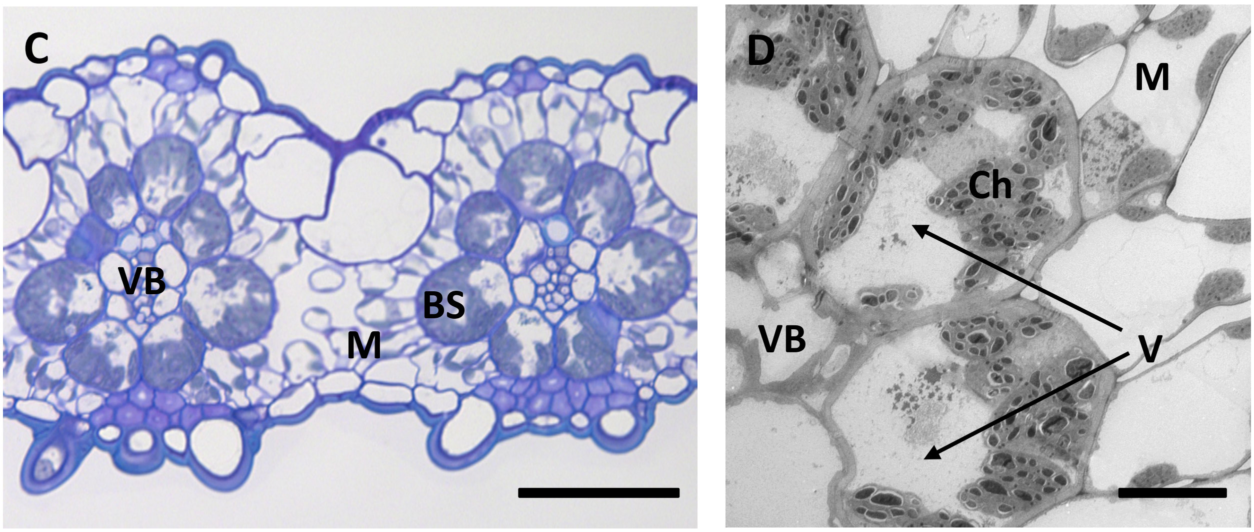

Supplementary Table S1. List of Zoysieae species studied, source of seeds, and GBIF records used in analyses of habitat MAP and MAT.

N	Species name	Source of seeds (USDA accession number or collection area)	Number of GBIF records	GBIF citation
1	*Sporobolus airoides* (Torr.) Torr.	W6 44471<br>W6 35350	328	GBIF.org (19 November 2023) GBIF Occurrence Download https://doi.org/10.15468/dl.6gg698
2	*Sporobolus agrostoides* Chiov (under syn. name *S. filipes)*	PI 209189	Not available	
3	*Sporobolus anglicus* (C.E. Hubb.) P.M. Peterson & Saarela *(*former *Spartina anglica)*	collected at Livingston Bay, WA, USA	20572	GBIF.org (19 November 2023) GBIF Occurrence Download https://doi.org/10.15468/dl.bgsxa3
4	*Sporobolus contractus* Hitchc.	PI 241073	82	GBIF.org (19 November 2023) GBIF Occurrence Download https://doi.org/10.15468/dl.9d7kw4
5	*Sporobolus cryptandrus* (Torr.) A. Gray	PI 676246	789	GBIF.org (19 November 2023) GBIF Occurrence Download https://doi.org/10.15468/dl.b46x9y
6	*Sporobolus fimbriatus* (Trin.) Nees	PI 185576	27	GBIF.org (19 November 2023) GBIF Occurrence Download https://doi.org/10.15468/dl.v3bhmu
7	*Sporobolus giganteus* Nash	W6 44479	16	GBIF.org (19 November 2023) GBIF Occurrence Download https://doi.org/10.15468/dl.xrezvd
8	*Sporobolus helvolus* (Trin.) T. Durand & Schinz	PI 219620	175	GBIF.org (19 November 2023) GBIF Occurrence Download https://doi.org/10.15468/dl.x8cvpz
9	*Sporobolus hookerianus* P.M. Peterson & Saarela *(*former *Spartina gracilis)*	W6 29998	108	GBIF.org (19 November 2023) GBIF Occurrence Download https://doi.org/10.15468/dl.5hgycn
10	*Sporobolus indicus* (L.) R.Br.	PI 203864	14865	GBIF.org (19 November 2023) GBIF Occurrence Download https://doi.org/10.15468/dl.9wtudr
11	*Sporobolus iocladus* (Nees ex Trin.) Nees	collected in Pakistan, provided by Dr.	Not available	

		Salman Gulzar (University of Karachi)		
12	*Sporobolus nitens* Stent	PI 208147	13	GBIF.org (19 November 2023) GBIF Occurrence Download https://doi.org/10.15468/dl.jgus9y
13	*Sporobolus phyllotrichus* Hochst.	PI 226098	Not available	
14	*Sporobolus texanus* Vasey	PI 478836	3	GBIF.org (19 November 2023) GBIF Occurrence Download https://doi.org/10.15468/dl.nf5ukb
15	*Sporobolus tremulus* (Willd.) Kunth	collected in Pakistan, provided by Dr. Salman Gulzar (University of Karachi)	Not available	
16	*Sporobolus wrightii* Munro ex Scribn.	W6 54414	99	GBIF.org (19 November 2023) GBIF Occurrence Download https://doi.org/10.15468/dl.2z4tp3
17	*Urochondra setulosa* (Trin.) C.E. Hubb	collected in Pakistan, provided by Dr. Salman Gulzar (University of Karachi)	6	GBIF.org (19 November 2023) GBIF Occurrence Download https://doi.org/10.15468/dl.gjad7c
18	*Zoysia matrella* (L.) Merr.	PI 231146	350	GBIF.org (19 November 2023) GBIF Occurrence Download https://doi.org/10.15468/dl.qk89w2
19	*Zoysia japonica* Steud.	PI 231389	810	GBIF.org (19 November 2023) GBIF Occurrence Download https://doi.org/10.15468/dl.hqamdq

Supplementary Table S2. Leaf anatomical traits related to photosynthesis. BS, bundle sheath; SDaba, abaxial stomata density; SDada, adaxial stomata density; CW, cell wall; M, mesophyll. NAD-ME species are marked by orange, PEP-CK species are marked by blue. Different letters indicate significant differences between species in a column at P≤0.05.

Species	Leaf thickness (minimal)*, µm	SDaba, # mm^{-2}	SDada, # mm^{-2}	Stomata size, abaxial, µm	Stomata size, adaxial, µm	CW thickness	
						BS, µm	M, µm
S. airoides	180.8±4.46a	102.3±6.27h	140.1±10.16d	31.6±0.82a	20.6±0.64b	1.05±0.05a	0.1±0.002b
S. contractus	117.1±1.84c	181.4±8.21ef	208.6±3.93c	22.7±0.38b	22.6±0.25b	0.82±0.03b	0.16±0.01a
S. cryptandrus	95.8±4.08de	85.7±5.10i	125.2±2.59e	30.9±0.61a	28.6±0.75a	0.35±0.01de	0.10±0.01c
S. giganteus	131.4±2.52c	105.3±4.11h	130.7±5.17e	30.7±0.45a	31.0±0.35a	0.47±0.06d	0.08±0.004d
S. helvolus	132.3±5.46bc	194.5±8.02de	170.7±6.26d	17.6±0.66b	16.2±0.66c	0.29±0.02e	0.06±0.003e
S. ioclados	201.0±4.62a	110.8±3.38h	141.5±2.53e	27.7±0.54a	23.5±0.36b	0.39±0.01d	0.096±0.002
S. phyllotrichus	81.4±1.96e	119.7±8.43gh	209.1±9.17c	18.1±0.68b	18.2±0.41c	0.96±0.02a	0.16±0.01a
S. texanus	130.2±3.24b	222.8±14.46c	291.5±11.46b	22.6±0.40b	21.8±0.36b	0.63±0.02c	0.11±0.01c
S. tremulus	103.5±2.41d	156.1±12.92f	313.9±10.05b	17.9±0.21b	15.8±0.46	0.88±0.03b	0.16±0.01a
S. wrightii	88.6±2.29e	180.4±6.50ef	187.2±4.25c	23.8±0.50b	23.4±0.31b	0.41±0.02d	0.09±0.002cd
U. setulosa	122.2±3.53bc	341.4±5.65a	168.6±2.53d	21.0±0.38b	22.2±0.51b	0.7±0.02c	0.08±0.02d
Average for NAD-ME species	125.9ns	163.7ns	189.7ns	24.1ns	22.2ns	0.63ns	0.11ns
S. anglicus	131.0±4.51bc	0.7±0.01j	168.4±5.23d	27.6±0.39a	32.4±0.38a	0.75±0.02bc	0.09±0.003c
S. agrostoides	91.2±2.23e	161.9±11.15f	127.5±4.82e	25.3±0.76ab	24.1±0.56ab	0.63±0.01c	0.15±0.01a

S. fimbriatus	104.7±3.23d	160.7±9.83f	190.5±15.27c	26.2±0.47a	17.4±0.31bc	0.71±0.02c	0.14±0.00bc
S. hookerianus	110.5±2.23cd	129.1±6.32g	160.1±6.62d	27.0±0.50a	20.5±0.56b	0.6±0.02c	0.12±0.02c
S. indicus	86.8±1.40e	100.1±2.21h	140.1±11.58de	29.2±0.46a	24.8±0.39a	0.75±0.02bc	0.11±0.01c
S. nitens	87.1±3.23e	101.0±2.41hi	133.5±6.51e	27.6±0.50a	24.3±0.25a	0.43±0.01d	0.16±0.01ab
Z. japonica	91.7±1.81e	203.7±5.62cd	410.9±11.21a	16.9±0.41c	13.2±0.30d	0.48±0.02d	0.11±0.01c
Z. matrella	86.8±2.69e	270.5±8.70b	455.3±11.03a	18.2±0.25bc	15.6±0.24cd	0.44±0.01d	0.17±0.01a
Average for PEP-CK species	115.0	154.6	203.2	24.3	21.9	0.62	0.12

*Leaf thickness measured between vascular bundles on leaf cross section

ns indicates non-significant differences between NAD-ME and PEP-CK groups at $p > 0.1$

Supplementary Table S3. List of Zoysieae species with suggested C_4 biochemical subtype based on different methods with references. NAD-ME species are marked by orange, PEP-CK species are marked by blue.

	Zoysieae species	Biochemical subtype	References for subtype description based on		
			All three decarboxylases: activity or Westerns	Only PEP-CK – activity, or Western, or transcripts, or *pck* gene	Anatomy
1	*Sporobolus airoides*	NAD-ME	[4] **Current study**		**Current study**
2	*Sporobolus africanus*	PEP-CK	[16]	[36]	[37]
3	*Sporobolus agrostoides (syn. S. filipes)*	PEP-CK	**Current study**		**Current study**
4	*Sporobolus alterniflorus* (former *Spartina alterniflora)*	PEP-CK	[9]		[25]
5	*Sporobolus anglicus*	PEP-CK	[9,12] **Current study**	[36]	[25] **Current study**
6	*Sporobolus bakeri* (former *Spartina bakeri)*	PEP-CK			[25]
7	*Sporobolus caroli*	NAD-ME	[16]		[37]
8	*Sporobolus contractus*	NAD-ME	**Current study**		**Current study**
9	*Sporobolus creber*	PEP-CK	[16]		[37]
10	*Sporobolus cryptandrus*	NAD-ME	[4] **Current study**		**Current study**
11	*Sporobolus cynosuroides* (former *Spartina cynosuroides)*	PEP-CK			[25]
12	*Sporobolus densiflorus* (former *Spartina densiflora)*	PEP-CK			[25]
13	*Sporobolus elongatus*	PEP-CK	[16]		[37]
14	*Sporobolus festivus*	PEP-CK		[36]	[26]
15	*Sporobolus fimbriatus*	PEP-CK	[5] **Current study**		**Current study**
16	*Sporobolus foliosus* (former *Spartina foliosa)*	PEP-CK			[25]
17	*Sporobolus giganteus*	NAD-ME	**Current study**		**Current study**
18	*Sporobolus helvolus*	NAD-ME	**Current study**		[26] **Current study**
19	*S. hookerianus* (former *Spartina gracilis)*	PEP-CK	**Current study**		[25] **Current study**
20	*Sporobolus indicus*	PEP-CK	**Current study**	[28]	**Current study**
21	*Sporobolus iocladus (=Sporobolus arabicus)*	NAD-ME	**Current study**		**Current study**
22	*Sporobolus jacquemontii*	PEP-CK	[16]	[28]	[37]

23	*Sporobolus maritimus* (former *Spartina maritima)*	PEP-CK		[36]	
24	*Sporobolus michauxianus* (former *Spartina pectinate)*	PEP-CK			[25]
25	*Sporobolus microprotus*	NAD-ME			[26]
26	*Sporobolus nitens*	PEP-CK	**Current study**		**Current study**
27	*Sporobolus paniculatus*	PEP-CK			[26]
28	*Sporobolus pectinellus*	PEP-CK			[26]
29	*Sporobolus phyllotrichus*	NAD-ME	**Current study**		**Current study**
30	*Sporobolus poiretii*	PEP-CK	[4]	[38]	
31	*Sporobolus pulchellus*	NAD-ME	[16]		[37]
32	*Sporobolus pumilus* (former *Spartina patens)*	PEP-CK			[25]
33	*Sporobolus pyramidalis*	PEP-CK		[28]	
34	*Sporobolus spartinus* (former *Spartina spartinae =Spartina argentinensis)*	PEP-CK			[25]
35	*Sporobolus stapfianus*	PEP-CK			[27]
36	*Sporobolus texanus*	NAD-ME	**Current study**		**Current study**
37	*Sporobolus tremulus*	NAD-ME	**Current study**		**Current study**
38	*Sporobolus virginicus*	NAD-ME			[29]
39	*Sporobolus wrightii*	NAD-ME	**Current study**		**Current study**
40	*Urochondra setulosa*	NAD-ME	[12] **Current study**		**Current study**
41	*Zoysia japonica*	PEP-CK	[4] **Current study**	[36]	**Current study**
42	*Zoysia matrella*	PEP-CK	**Current study**		**Current study**

References

4. Gutierrez, M.; Gracen, V.E.; Edwards, G.E. Biochemical and cytological relationships in C_4 plants. *Planta* **1974**, *119*, 279-300. https://doi.org/10.1007/BF00388331
5. Hatch, M.D.; Kagawa, T.; Craig, S. Subdivision of C_4-pathway species based on differing C_4 acid decarboxylating systems and ultrastructural features. *Australian Journal of Plant Physiology* **1975**, *2*, 111-128. https://doi.org/10.1071/PP9750111.
9. Voznesenskaya, E.V.; Franceschi, V.R.; Chuong, S.D.X.; Edwards, G.E. Functional characterization of phosphoenolpyruvate carboxykinase type C_4 leaf anatomy: Immuno, cytochemical and ultrastructural analyses. *Annals of Botany* **2006**, *98*, 77-91. https://doi.org/10.1093/aob/mcl096.
12. Koteyeva, N.K.; Voznesenskaya, E.V.; Edwards, G.E. An assessment of the capacity for phosphoenolpyruvate carboxykinase to contribute to C_4 photosynthesis. *Plant Science* **2015**, *235*, 70-80. http://dx.doi.org/10.1016/j.plantsci.2015.03.004.
16. Prendergast, H.D.V.; Hattersley, P.W.; Stone, N.E. New structural/biochemical associations in leaf blades of C_4 grasses (Poaceae). *Australian Journal of Plant Physiology* **1987**, *14*, 403-420. https://doi.org/10.1071/PP9870403.
25. Maricle, B.R.; Koteyeva, N.K.; Voznesenskaya, E.V.; Thomasson, J.R.; Edwards, G.E. Diversity in leaf anatomy, and stomatal distribution and conductance, between salt marsh and freshwater species in

the C_4 genus *Spartina* (Poaceae). *New Phytologist* **2009**, *184*, 216-233. https://doi.org/10.1111/j.1469-8137.2009.02903.x.
26. Schmidt, M.; König, K.; Müller, J.; Brunken, U.; Zizka, G. Modelling the distribution of photosynthetic types of grasses in Sahelian Burkina Faso with high-resolution satellite data. *Ecotropica* **2011**, *17*, 53-63.
27. Vecchia, F.D.; El Asmar, T.; Calamassi, R.; Rascio, N.; Vazzana, C. Morphological and ultrastructural aspects of dehydration and rehydration in leaves of *Sporobolus stapfianus*. *Plant Growth Regulation* **1998**, *24*, 219-228. https://doi.org/10.1023/A:1005853527769.
28. Walker, R.P.; Acheson, R.M.; Tecsi, L.I.; Leegood, R.C. Phospho*enol*pyruvate carboxykinase in C_4 plants: its role and regulation. *Australian Journal of Plant Physiology* **1997**, *24*, 459-468. https://doi.org/10.1071/PP97007.
29. Kemp, P.R.; Cunningham, G.L.; Adams, H.P. Specialization of mesophyll morphology in relation to C_4 photosynthesis in the Poaceae. *American Journal of Botany* **1983**, *70*, 349-354. https://doi.org/10.2307/2443242.
36. Christin, P.-A.; Petitpierre, B.; Salamin, N.; Büchi, L.; Besnard, G. Evolution of C_4 phosphoenolpyruvate carboxykinase in grasses, from genotype to phenotype. *Molecular Biology and Evolution* **2009**, *26*, 357-365. https://doi.org/10.1093/molbev/msn255.
37. Prendergast, H.D.V.; Hattersley, P.W. Australian C_4 grasses (Poaceae): leaf blade anatomical features in relation to C_4 acid decarboxylation types. *Australian Journal of Botany* **1987**, *35*, 355-382. https://doi.org/10.1071/BT9870355.
38. Edwards, G.E.; Kanai, R.; Black, C.C. Phosphoenolpyruvate carboxykinase in leaves of certain plants which fix CO_2 by the C_4-dicarboxylic acid cycle of photosynthesis. *Biochemical and Biophysical Research Communications* **1971**, *45*, 278-285. https://doi.org/10.1016/0006-291X(71)90814-X.

Supplementary Table S4. Sporobolus accessions used for phylogeny reconstruction

NCBI nucleotide	RpL32 and rpl32-trnL	ndhA	rps16	rps16-trnK
Eleusine indica voucher Peterson 21362, Saarela & Flores Villegas	GU359797.1	GU359473.1	GU360472.1	GU360496.1
Eragrostiella leioptera voucher Chand 7961	GU359827.1	GU359486.1	JQ345280.1	GU360529.1
Gymnopogon grandiflorus voucher Peterson 16642 & Refulio-Rodriguez	GU359816.1	GU359436.1	GU360383.1	GU360581.1
Sporobolus africanus voucher Peterson 24024	KM010735	KM010567	KM010955	KM011160
Sporobolus africanus voucher Peterson 24121	KM010736	KM010568	KM010956	KM011161
Sporobolus airoides subsp. airoides voucher Peterson 24587 & Romaschenko	KM010738	KM010570	KM010958	KM011163
Sporobolus airoides subsp. airoides voucher Peterson 24853 & Romaschenko	KM010739	KM010571	KM010959	KM011164
Sporobolus airoides voucher Peterson 10002, Annable & Valdes-Reyna	KM010737	KM010569	KM010957	KM011162
Sporobolus alterniflorus voucher Naylov 236	KM010701.1	KM010540.1	KM010924.1	KM011128.1
Sporobolus anglicus voucher Matthews s.n.	KM010702	KM010541	KM010925	KM011129
Sporobolus anglicus voucher Williams 2004-1	KM010703	KM010542	KM010926	KM011130
Sporobolus anglicus voucher Williams 2004-2	KM010704	KM010543	KM010927	KM011131
Sporobolus bakeri voucher Killip 44361	KM010705.1	KM010544.1	KM010928.1	KM011132.1
Sporobolus carolii voucher Saarela 1626, Peterson & Soreng	KM010759.1	KM010584.1	KM010976.1	-
Sporobolus carolii voucher Speak 1915	KM010760.1	KM010585.1	KM010977.1	KM011183.1
Sporobolus contractus voucher Perez 196	KM010772	–	KM010988	KM011194
Sporobolus creber voucher Brown 498	KM010779.1	KM010600.1	KM010994.1	KM011200.1
Sporobolus cryptandrus voucher Peterson 24454 & Romaschenko	KM010780	KM010601	KM010995	KM011201
Sporobolus cryptandrus voucher Peterson 24485 & Romaschenko	KM010781	KM010602	KM010996	KM011202
Sporobolus cynosuroides voucher Fisher 33123	KM010708.1	KM010545.1	KM010930.1	KM011135.1
Sporobolus cynosuroides voucher Hill 15630	KM010709.1	KM010546.1	KM010931.1	KM011136.1
Sporobolus densiflorus voucher Clayton 4723 & Eiten	KM010717.1	KM010553.1	KM010939.1	KM011144.1
Sporobolus densiflorus voucher Lomer 5723	-	KM010547.1	KM010932.1	KM011137.1
Sporobolus festivus voucher Peterson 23853	KM010791	KM010608	KM011004	KM011209
Sporobolus fimbriatus voucher Peterson 24206, Soreng & Romaschenko	KM010793	KM010609	KM011005	KM011210
Sporobolus fimbriatus voucher Peterson 24241, Soreng & Romaschenko	KM010794	KM010610	KM011006	KM011211
Sporobolus fimbriatus voucher Peterson 24280, Soreng & Romaschenko	–	KM010611	KM011007	KM011212
Sporobolus foliosus voucher Reeder 6652 & Reeder	KM010711.1	KM010548.1	KM010933.1	KM011138.1
Sporobolus giganteus voucher Page 2628	KM010800	–	KM011011	KM011216
Sporobolus helvolus voucher Laegaard 17063 & Traore	KM010802	–	KM011013	KM011217

Sporobolus helvolus voucher Peterson 24217, Soreng & Romaschenko	KM010803	KM010615	KM011014	KM011218
Sporobolus hookerianus voucher Hendrickson 41	KM010712	KM010549	KM010934	KM011139
Sporobolus hookerianus voucher Scoggan 15626	KM010714	KM010551	KM010936	KM011141
Sporobolus indicus var. pyramidalis voucher Peterson 24150	KM010858	KM010646	KM011062	KM011261
Sporobolus indicus var. pyramidalis voucher Peterson 24150, Soreng & Romaschenko	KM010858.1	KM010646.1	-	KM011261.1
Sporobolus indicus var. pyramidalis voucher Senaratne E6082-11	KM010859	KM010647	KM011063	KM011262
Sporobolus indicus var. pyramidalis voucher Senaratne E6082-11	KM010859.1	KM010647.1	KM011063.1	KM011262.1
Sporobolus indicus voucher Peterson 22025 & Saarela	GU359913	GU359504	GU360355	GU360630
Sporobolus indicus voucher Peterson 7337	KM010806	KM010617	KM011017	KM011221
Sporobolus ioclados voucher Smook 5920	KM010808	KM010619	KM011019	KM011223
Sporobolus jacquemontii voucher Estrada 18964 et al.	KM010809	KM010620	KM011020	KM011224
Sporobolus jacquemontii voucher Peterson 15902 & Valdes-Reyna	KM010810	KM010621	KM011021	KM011225
Sporobolus maritimus voucher Fernandez Casas 5537, Castroviejo, Munoz Garmendia & Susanna	KM010715.1	KM010552.1	KM010937.1	KM011142.1
Sporobolus maritimus voucher Marchant s.n.	KM010716.1	-	KM010938.1	KM011143.1
Sporobolus michauxianus voucher Cooperrider s.n.	KM010722.1	KM010556.1	KM010943.1	KM011149.1
Sporobolus michauxianus voucher Dirig 2812	KM010723.1	KM010557.1	KM010944.1	KM011150.1
Sporobolus microprotus voucher Laegaard 17894 & Traore	KM010824	KM010629	KM011035	KM011235
Sporobolus microprotus voucher Laegaard 17894 & Traore	KM010824.1	KM010629.1	KM011035.1	-
Sporobolus nitens voucher Laegaard 15893	KM010837	KM010634	KM011043	KM011246
Sporobolus pectinellus voucher Peterson 23978, Soreng & Romaschenko	KM010848.1	KM010641.1	KM011052.1	KM011252.1
Sporobolus phyllotrichus voucher Greenway 11844 & Kanuri	KM010851	–	KM011055	–
Sporobolus pumilus voucher Dutton 2536	KM010719.1	KM010554.1	KM010940.1	KM011146.1
Sporobolus pumilus voucher Peterson 24435 & Romaschenko	KM010720.1	-	KM010941.1	KM011147.1
Sporobolus pumilus voucher Shchepanek 6426 & Dugal	KM010721.1	KM010555.1	KM010942.1	KM011148.1
Sporobolus spartinus voucher Reeder 4568 & C. Reeder	KM010724.1	KM010558.1	KM010945.1	KM011151.1
Sporobolus spartinus voucher Villarreal 3201	KM010725.1	KM010559.1	KM010946.1	KM011152.1
Sporobolus stapfianus voucher Laegaard 15939	KM010882.1	-	KM011084.1	KM011283.1
Sporobolus texanus voucher Churchill 2645 & Kaul	GU359908	GU359509	GU360376	GU360626
Sporobolus virginicus voucher Peterson 23820, Soreng & Romaschenko	KM010900.1	-	KM011102.1	KM011299.1
Sporobolus virginicus voucher US:Peterson 15683 & Soreng	GU359892.1	GU359502.1	GU360362.1	GU360610.1
Sporobolus wrightii voucher Peterson 10638 & Annable	KM010902	KM010677	KM011104	KM011301
Sporobolus wrightii voucher Peterson 19841 & Lara-Contreras	GU359906	GU359511	GU360348	GU360624
Sporobolus wrightii voucher Peterson 24841 & Romaschenko	KM010903	KM010678	KM011105	KM011302

Tripogon multiflorus voucher Spellenberg 7441	JQ345360.1	JQ345232.1	JQ345315.1	JQ345274.1
Tripogon trifidus voucher Laegaard 21668 & Norsangsri	KP873641.1	KP873957.1	KP874094.1	KP873822.1
Urochondra setulosa voucher Bailey 10868	KM010906.1	KM010679.1	KM011106.1	KM011305.1
Urochondra setulosa voucher Baldini s.n.	KM010907.1	KM010680.1	KM011107.1	KM011306.1
Urochondra setulosa voucher Inckennon 181	KM010908.1	KM010681.1	KM011108.1	KM011307.1
Urochondra setulosa voucher Rechinger 27496	KM010909.1	KM010682.1	KM011109.1	KM011308.1
Zoysia japonica voucher US:Kuragadake s.n.	GU359923.1	GU359547.1	-	GU360643.1
Zoysia macrantha subsp. walshii voucher US:Loch 435	GU359922.1	GU359548.1	GU360345	GU360642.1
Zoysia macrantha voucher US:Soreng 5913 & Peterson	GU360017.1	GU359558.1	GU360346.1	GU360641.1
